# Evaluating the correspondence of neuropsychological profiles with clinical diagnostic criteria for frontotemporal lobar degeneration and Alzheimer's disease: insights from *k*-means clustering

**DOI:** 10.3389/frdem.2026.1757680

**Published:** 2026-08-31

**Authors:** Marie Söntgerath, Simone Cutini, Angelika I. T. Thöne-Otto, Annerose Engel, Frank Regenbrecht, Maryna Polyakova, Karsten Mueller, Nico Scherf, Fabiola Boehm, Sarah Anderl-Straub, Bernhard Landwehrmeyer, Adrian Danek, Janine Diehl-Schmid, Klaus Fassbender, Klaus Fliessbach, Holger Jahn, Holger Kornhuber, Martin Lauer, Johannes Prudlo, Matthis Synofzik, Jens Wiltfang, Markus Otto, Matthias L. Schroeter

**Affiliations:** 1 Department of Neurology, Max Planck Institute for Human Cognitive and Brain Sciences, Leipzig, Germany; 2 Department of Cognitive Neurology, University Clinic, University of Leipzig, Leipzig, Germany; 3 Department of Neurology, University Clinic, Martin Luther University Halle-Wittenberg, Halle (Saale), Germany; 4 Department of Developmental Psychology, University of Padova, Padua, Italy; 5 Department of Neurology, Charles University, First Faculty of Medicine and General University Hospital, Prague, Czechia; 6 Department of Neurology, University Clinic, University Ulm, Ulm, Germany; 7 Department of Neurology, University Clinic, Ludwig-Maximilians-University Munich, Munich, Germany; 8 German Center for Neurodegenerative Diseases (DZNE), Research Site Munich, Munich, Germany; 9 School of Medicine, Department of Psychiatry and Psychotherapy, Technical University of Munich, Munich, Germany; 10 kbo-Inn-Salzach-Klinikum, Clinical Center for Psychiatry, Psychotherapy, Psychosomatic Medicine, Geriatrics and Neurology, Wasserburg am Inn, Germany; 11 Department of Neurology, Saarland University, Homburg, Germany; 12 Department of Psychiatry and Psychotherapy, University of Bonn, Bonn, Germany; 13 Department of Psychiatry and Psychotherapy, University Clinic Hamburg-Eppendorf, Hamburg, Germany; 14 Department of Psychiatry and Psychotherapy, University Clinic, Friedrich-Alexander University Erlangen-Nürnberg, Erlangen, Germany; 15 Department of Psychiatry, Psychosomatic and Psychotherapy, University of Würzburg, Würzburg, Germany; 16 Department of Neurology, Rostock University Medical Center, Rostock, Germany; 17 German Center for Neurodegenerative Diseases (DZNE), Rostock-Greifswald, Rostock, Germany; 18 Department of Neurodegeneration, Hertie Institute for Clinical Brain Research (HIH), University of Tübingen, Tübingen, Germany; 19 Department of Psychiatry and Psychotherapy, University Medical Center Göttingen, Göttingen, Germany; 20 German Center for Neurodegenerative Diseases (DZNE), Göttingen, Germany; 21 Department of Medical Sciences, Neurosciences and Signaling Group, Institute of Biomedicine (iBiMED), University of Aveiro, Aveiro, Portugal

**Keywords:** Alzheimer's dementia, differential diagnosis, frontotemporal dementia, frontotemporal lobar degeneration, *K*-means clustering, neuropsychological tests, primary progressive aphasia

## Abstract

**Introduction:**

Diagnosis of frontotemporal lobar degeneration (FTLD) is complicated by high heterogeneity within diagnostic groups and overlap between them. Using an unsupervised machine learning technique this study aimed to increase understanding of neuropsychological profiles in the spectrum of FTLD syndromes and to assess the consistency of these neuropsychological profiles with diagnostic criteria.

**Methods:**

Data from the German FTLD Consortium (FTLD-c) including behavioral variant of frontotemporal dementia (bvFTD), primary progressive aphasia (PPA) syndromes, i.e., semantic variant (svPPA), and non-fluent variant (nfvPPA), healthy controls, and amnestic Alzheimer's disease (AD), and logopenic variant (lvPPA) was used. *K*-means clustering was applied on neuropsychological and behavioral data. Analysis was repeated for varying cluster numbers (*k* = 2–9). Resulting clusters were inspected on the correspondence with current diagnostic groups and neuropsychological profiles of the clusters were explored.

**Results:**

The results indicated that, based on neuropsychological profiles, particularly homogeneous clusters emerged for healthy controls, svPPA and bvFTD. In contrast, amnestic AD did not separate well from other diagnostic groups, and nfvPPA and lvPPA clustered together. Inspecting the neuropsychological profiles of the clusters showed that segregation of bvFTD from other patient groups was most supported by companion-rated questionnaires focused on behavior. Patients with svPPA showed impairment in both language and verbal memory tasks. LvPPA and nfvPPA showed impairment in processing speed as well as short term and working memory.

**Discussion:**

Using clustering this study provides insight into data-driven grouping of participants with FTLD based on neuropsychological deep-phenotyping. Whereas our approach confirmed svPPA and bvFTD to have comparatively homogeneous neuropsychological profiles, results suggest that the non-fluent PPA syndromes–lvPPA and nfvPPA–are difficult to segregate clinically, at least with the tests applied in our cohort. Our findings may suggest the need for further specification of clinical diagnostic criteria for these diseases.

## 1 Introduction

Dementia is a clinical syndrome characterized by progressive cognitive decline that can manifest in different ways, depending on the underlying pathology and the brain areas affected. Frontotemporal lobar degeneration (FTLD) is the second most common cause for early-onset dementia, following amnestic Alzheimer's disease (AD) (Bang et al., 2015; Rascovsky et al., 2011; Gorno-Tempini et al., 2011). Here, we use FTLD as the umbrella term for the clinical syndromes associated with FTLD. The pre-dominant clinical phenotype of FTLD is behavioral variant frontotemporal dementia (bvFTD). BvFTD is characterized by changes in behavior, most commonly involving apathy and disinhibition but also mental rigidity and loss of empathy. With progression of the disease, dietary changes, such as overeating, and decreased hygiene are observed (Bang et al., 2015). Additional to behavioral changes, diagnostic criteria include as supportive features executive dysfunction with relatively intact memory and visuospatial skills (Rascovsky et al., 2011).

Beyond changes in personality and behavior, FTLD can also manifest as language-predominant dementias, known as primary progressive aphasias (PPA). Three main PPA variants have been described. The non-fluent variant PPA (nfvPPA) is characterized by effortful speech due to speech apraxia or agrammatism making the speech seem telegraphic (Gorno-Tempini et al., 2011; Warren et al., 2013). Over time, patients may develop complete mutism (Grossman, 2010) while comprehension and semantic knowledge are usually spared. In contrast, core symptoms of the semantic variant PPA (svPPA) are loss of semantic knowledge resulting in problems in confrontation naming (i.e., anomia) and word comprehension (Gorno-Tempini et al., 2011; Henry and Grasso, 2018). Speech typically remains fluid while becoming progressively empty in content, reducing to platitudes. During reading, patients may make regularization errors in pronunciation due to loss of associated meaning (Macoir et al., 2021).

A third variant of PPA, the logopenic variant (lvPPA), is commonly considered an atypical AD, as it has been related to underlying AD pathology (Irwin et al., 2013). The main characteristic of lvPPA is disturbed phonemic processing leading to phonological errors in both spontaneous speech and structured tasks of repetition or naming (Gorno-Tempini et al., 2011). Difficulties in repetition may be intensified by phonological short-term memory deficits, with greater impairments for longer sentences (Gil-Navarro et al., 2013). Phonological dyslexia and dysgraphia are present for both words and non-words (Henry and Grasso, 2018).

In clinical practice, correct differential diagnosis of the FTLD syndromes remains difficult often requiring more than 3 years (Beber and Chaves, 2013; van Vliet et al., 2013), particularly in young-onset cases. Due to changes in personality and behavior, a considerable proportion of patients may initially receive a diagnosis of a primary psychiatric disorder (Woolley et al., 2011). For example, apathetic symptoms or changes in eating behavior have been mentioned as reasons for confusing bvFTD with major depressive disorder. Additional challenges arise due to significant heterogeneity within (Beber and Chaves, 2013; Harris et al., 2013) and considerable overlap between neuropsychological profiles of the dementias described (Bang et al., 2015; Coyle-Gilchrist et al., 2016; Kamath et al., 2019). Deficits in episodic memory function, which are considered a hallmark of amnestic AD, may be more pronounced in FTLD syndromes than previously thought (Hornberger and Piguet, 2012). In bvFTD retrieving problems may be related to executive dysfunction, while memory problems in lvPPA may be due to impaired phonological loop functioning (Hornberger and Piguet, 2012; Piguet et al., 2011; Foxe et al., 2021). Additionally, correct differential diagnosis may be hampered by behavioral symptoms observed in svPPA, such as mental rigidity and loss of empathy, which may resemble those observed in bvFTD (Bang et al., 2015). Lastly, nfvPPA and lvPPA may be particularly likely to be confounded due to similarity in slowed, halting speech (Bürger et al., 2017; Gorno-Tempini et al., 2008; Harris et al., 2019).

As a consequence of diagnostic difficulties observed in clinical settings, calls for revisions of diagnostic criteria have been expressed (Hornberger and Piguet, 2012; Piguet et al., 2011; Harris et al., 2019; Sajjadi et al., 2012). Previous articles have shown that a considerable proportion of patients with a PPA may not meet the diagnostic criteria for one of the three variants and the existence of a logopenic variant has been questioned (Harris et al., 2013; Sajjadi et al., 2012). Concerning bvFTD, disturbances in executive function may not be specific to differentiate bvFTD from other dementia syndromes (Walker et al., 2005; Schroeter et al., 2014; Foran et al., 2021; Reul et al., 2017; Overbeek et al., 2020). More recent literature on bvFTD has shifted the focus toward the study of social and emotional functions (Schroeter et al., 2018; Gossink et al., 2018; Rankin, 2021). Addressing the gap of diagnostic precision is an ongoing objective. It is relevant to psychological and social wellbeing of both patients and caregivers (Musa et al., 2020; Weder et al., 2007) and is necessary to power research on therapy and treatment, especially in the age of new disease-specific drugs (van Dyck et al., 2023).

Given these challenges in differential diagnosis, novel computational approaches such as machine learning (ML) techniques may be promising to improve diagnostic accuracy. This is particularly advantageous in cases where multidimensional data is available and high levels of diagnostic uncertainty persist. By being inherently multivariate ML can aid in finding patterns within data rather than looking for single predictive tests (Yanase and Triantaphyllou, 2019). Unsupervised ML can be used to compute similarity of unlabeled data in a multidimensional space (Fan et al., 2020) in order to delineate subgroups or detect outliers in a data-driven way (Bhardwaj et al., 2017; Habes et al., 2020). By uncovering patient similarities and differences, unsupervised ML may bring new insights to the existing diagnostic challenges. For example, a previous study, applying unsupervised ML on neuroimaging data from patients diagnosed with a PPA, determined five clusters (Matias-Guiu et al., 2019). In turn, neuroimaging-based clustering studies in bvFTD indicated the presence of several subgroups with distinct atrophy patterns (Bruun et al., 2019; Ranasinghe et al., 2016). So far, applying ML on a breadth of neuropsychological variables from patients with FTLD has been rarely explored. Instead, studies have focused on data from language assessments and various neuroimaging techniques for PPA (Fan et al., 2020; Leyton et al., 2014) and bvFTD (Cerami et al., 2016; Josephs et al., 2009), respectively. Further, only few studies have evaluated multiclass analysis (e.g., Zimmerer et al., 2020; Kim et al., 2019) and those that have, often grouped FTLD phenotypes together to separate them from AD, Lewy body dementia, Parkinson's disease dementia or vascular dementia (Lin et al., 2020; Garn et al., 2017).

This study aims to address this gap, applying unsupervised ML to neuropsychological data from a large cohort of participants with diagnoses from the FTLD and AD spectrum. An analysis of deep-phenotyping neuropsychological data may yield new insights for each one of the patient groups as well as for their distinction. The main goal of the current study was to use a data-driven approach to investigate similarities in neuropsychological profiles of patients diagnosed with a FTLD type and to evaluate how well the results correspond to the current diagnostic criteria. For this purpose, *k*-means clustering, an unsupervised ML algorithm, was used to explore behavioral and neuropsychological data from patients diagnosed with bvFTD, svPPA, nfvPPA, lvPPA, amnestic AD and healthy controls. This exploratory analysis may inform differential diagnosis which persists to be difficult. Neuropsychological examinations being a standard part of diagnoses in memory clinics (Schild et al., 2023; Deuschl et al., 2016), results from this study may inform their refinement when differentiating dementias from the FTLD and AD spectrum.

Exploration of the data in this study followed three major goals: (1) Investigating correspondence of clusters with diagnostic groups; (2) Investigating neuropsychological profiles of the emerging clusters; and (3) Comparing the results with current diagnostic standards. To the best of our knowledge, this is the first study to analyze all five diagnostic groups (i.e., bvFTD, svPPA, nfvPPA, lvPPA, amnestic AD) using clustering approaches on neuropsychological data. Focus was put on the exploration of the following contrasts thought to be particularly difficult but clinically relevant: (1) bvFTD vs. amnestic AD, (2) the three PPA variants and (3) svPPA vs. bvFTD.

## 2 Methods

### 2.1 Patient data

**Figure 1:**
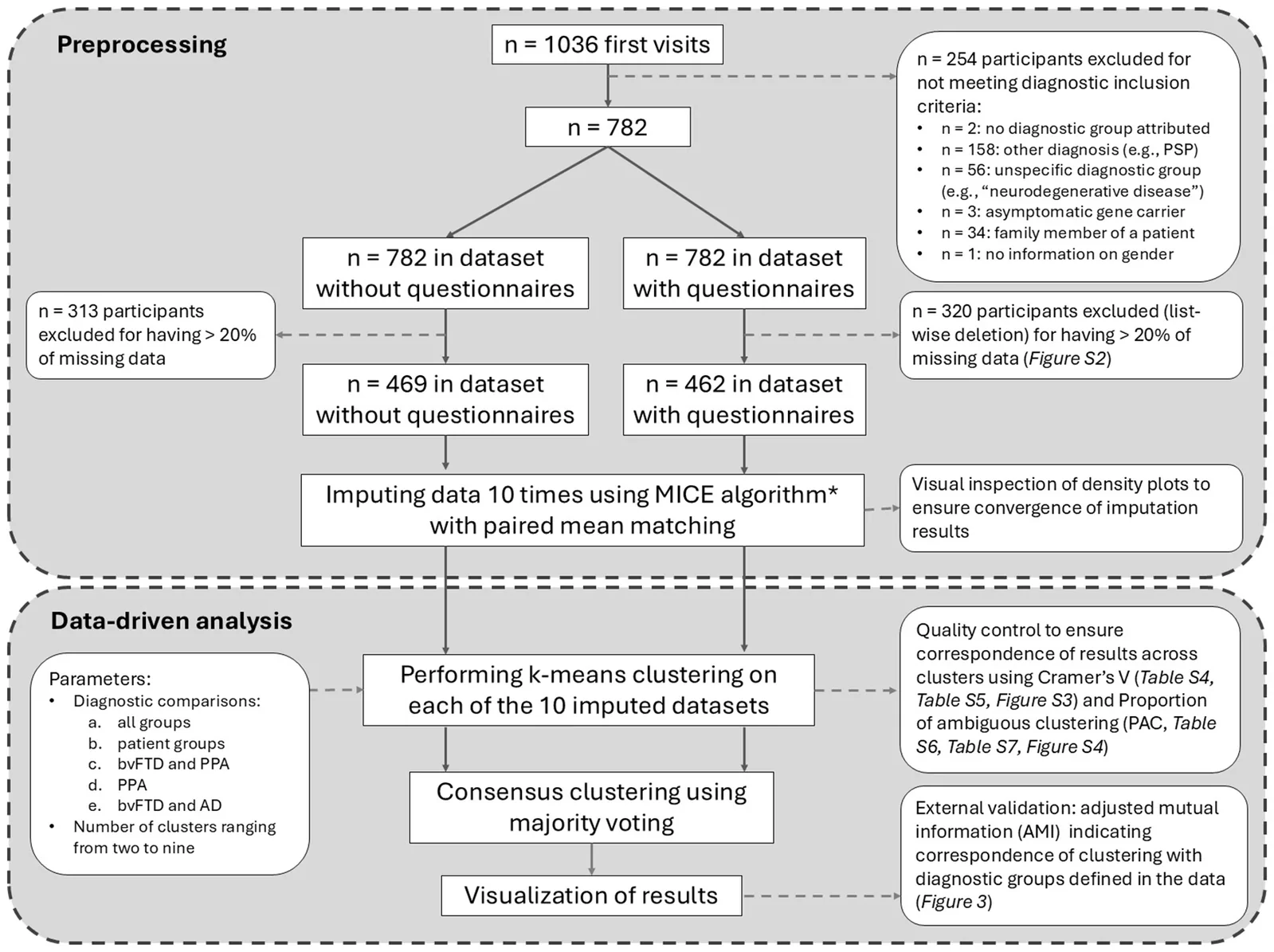
Flowchart of participant selection and analysis. *van Buuren and Groothuis-Oudshoorn (2011). AD, amnestic Alzheimer's Disease; bvFTD, behavioral variant frontotemporal dementia; MICE, multiple imputation by chained equations; PPA, primary progressive aphasia; PSP, progressive supranuclear palsy.

Patient data was provided by the German FTLD Consortium collected from 13 University clinics or research centers across Germany (Otto et al., 2011). We focused on data from the first participant visit to ensure early disease stage and proximity to diagnostic decision. From the 1,036 participants originally in the dataset, 254 were excluded for not belonging to one of the diagnostic groups of interest (Figure 1). In total 782 participants remained for further analysis. To address missing values, a combination of list-wise deletion and imputation was used. First, participants with more than 20% of missing variables were removed, thus leaving us with participants that had followed relatively complete visits. Next, remaining missing values were imputed using multiple imputation by chained equations (MICE) (van Buuren, 2019). The specific approach of handling missing data is detailed in the Supplementary materials.

The analysis was performed twice, firstly with neuropsychological and clinical data only, secondly additionally including questionnaires. The first analysis without questionnaires was focused on cognitive and language functions to investigate their potential for differential diagnosis. The second analysis took additionally behavioral and personality changes into account, which is particularly relevant for bvFTD. This allowed investigating the additional value of the behavioral questionnaires beyond cognitive assessments. Additionally, due to comparatively high levels of missingness in the behavioral questionnaires, robustness of results could be inspected by comparing them with the analysis without behavioral questionnaires. A total of 469 and 462 participants remained for the analysis without and with questionnaires, respectively (Table 1).

**Table 1:** Demographics and clinical characteristics for included groups and split by analysis excluding and including questionnaires.

Group	N	Total %	Male %	Mean age in years (SD)	Mean years of education in years (SD)	Mean FTLD-CDR SOB (SD)	Mean disease duration in years (SD)
Without questionnaires							
HC	49	10.4	44.9	64.5 (9.3)	15.0 (3.1)	0.1 (0.3)	—
Amnestic AD	74	15.8	54.1	66.1 (9.7)	13.5 (3.3)	5.8 (3.4)	2.8 (2.8)
bvFTD	171	36.5	62.6	62.3 (9.3)	13.5 (3.0)	6.8 (3.9)	3.4 (3.8)
svPPA	52	11.1	46.2	62.9 (7.9)	14.7 (3.2)	4.9 (2.4)	2.6 (1.9)
nfvPPA	76	16.2	48.7	68.9 (8.3)	13.1 (3.3)	4.2 (2.4)	1.9 (1.2)
lvPPA	47	10	48.9	68.7 (6.1)	13.3 (3.5)	4.7 (2.8)	4.0 (4.0)
Chi-square test			n. s.				
Kruskal-Wallis rank sum test				*X*^2^ = 43.299 *p* = 3.214e−08	*X*^2^ = 20.139 *p* = 0.001177	*X*^2^ = 158.79 *p* < 2.2e−16	*X*^2^ = 11.09 *p* = 0.02557
Pairwise comparison using Wilcoxon rank sum test (Bonferroni corrected)				bvFTD–nfvPPA^***^ bvFTD–lvPPA^***^ svPPA–nfvPPA^***^ svPPA–lvPPA^**^ bvFTD–AD^*^	HC–bvFTD^*^ HC–nfvPPA^*^	HC and all other groups^***^ bvFTD–nfvPPA^***^ bvFTD–lvPPA^**^ bvFTD–svPPA^**^	nfvPPA–lvPPA^*^
With questionnaires							
HC	49	10.6	44.9	64.5 (9.3)	15.0 (3.1)	0.1 (0.3)	—
Amnestic AD	70	15.2	52.9	66.4 (9.7)	13.4 (3.4)	5.7 (3.3)	2.8 (2.8)
bvFTD	173	37.4	62.4	62.3 (9.5)	13.4 (2.9)	6.9 (4.0)	3.4 (3.5)
svPPA	50	10.8	46.0	63.2 (7.9)	14.6 (3.3)	4.8 (2.4)	2.6 (1.9)
nfvPPA	72	15.6	47.2	68.7 (8.6)	13.1 (3.4)	4.2 (2.5)	2.0 (1.3)
lvPPA	48	10.4	47.9	68.4 (6.2)	13.3 (3.4)	4.7 (2.8)	4.0 (3.9)
Chi-square test			n. s.				
Kruskal-Wallis rank sum test				*X*^2^ = 39.28 *p* = 2.085e−07	*X*^2^ = 18.414 *p* = 0.00247	*X*^2^ = 159.14 *p* < 2.2e-16	*X*^2^ = 9.8962 *p* = 0.04221
Pairwise comparison using Wilcoxon rank sum test (Bonferroni corrected)				bvFTD–nfvPPA^***^ bvFTD–lvPPA^***^ svPPA–nfvPPA^**^ svPPA–lvPPA^**^ bvFTD–AD^*^	HC–bvFTD^*^ HC–nfvPPA^*^	HC and all other groups^***^ bvFTD–nfvPPA^***^ bvFTD–lvPPA^**^ bvFTD–svPPA^**^	nfvPPA–lvPPA^*^

AD, Alzheimer's disease; bvFTD, behavioral variant frontotemporal dementia; FTLD CDR SOB, frontotemporal lobar degeneration clinical dementia rating sum of boxes; HC, healthy control; lvPPA, logopenic variant primary progressive aphasia; nfvPPA, non-fluent variant primary progressive aphasia; svPPA, semantic variant primary progressive aphasia; n. s., non-significant; ^*^significant at *p* < 0.05, ^**^significant at *p* < 0.01, ^***^significant at *p* < 0.001.

The participants mean age varied between 62.3 and 68.9 years for patients with bvFTD and nfvPPA, respectively. As measure of disease severity, the sum of boxes of the clinical dementia rating (FTLD-CDR SOB) (Morris, 1993; Knopman et al., 2008) was used. As expected, healthy controls differed significantly on the FTLD-CDR SOB compared to all other groups. Additionally, the bvFTD group had significantly higher scores on the FTLD-CDR SOB compared to the three PPA variants. The mean disease durationat study visit ranged from 1.9 to 4.0 years in nfvPPA and lvPPA, respectively, thus indicating significantly later participation in the study in lvPPA compared to nfvPPA (Table 1).

Diagnostic decisions were made by experienced neurologists or psychiatrists based on comprehensive medical, neuropsychological and speech therapeutic examination. Medical information included brain imaging and degenerative markers in the cerebrospinal fluid assessed by lumbar puncture. Of the included participants diagnosed with bvFTD or PPA, 119 (34%) had a clinical, 204 (59%) additional image-supported, and 23 (7%) a neuropathologically confirmed diagnosis in the analysis excluding questionnaires. In the analysis including questionnaires, 124 (36%) patients with bvFTD or PPA had a clinical, 197 (57%) had additional image-supported and 22 (6%) a neuropathologically confirmed diagnosis. Diagnosis was defined according to established international diagnostic criteria (Rascovsky et al., 2011; Gorno-Tempini et al., 2011). Control groups included healthy participants and patients with amnestic AD, as defined by McKhann and colleagues (McKhann et al., 2011) including biomarker data. Participants provided written informed consent. The study was approved by the ethics committees of all contributing Universities (ethics committee University Leipzig ID 137–11–18042011 and University Ulm ID #39/11) and was in accordance with the latest version of the Declaration of Helsinki.

### 2.2 Neuropsychological test scores

Demographic variables included for further analysis were participants' age and years of education. The neuropsychological test scores used can broadly be attributed to the six neurocognitive domains defined in the DSM-5 aiming to cover the full spectrum of cognition (American Psychiatric Association, 2013). The FTLD-CDR with its eight sub-domains was used as a clinical staging instrument (Morris, 1993; Knopman et al., 2008). Further, the CERAD-NAB-Plus battery (Moms et al., 1989; Fillenbaum et al., 2008) developed for AD and standardly applied in German memory clinics (Schild et al., 2023) was used. The CERAD-NAB consists pre-dominantly of memory tests (besides BNT, verbal fluency and visuoconstruction), in accordance with the encoding deficits of episodic memory expected in amnestic AD. Additional tests were added specifically for the language, executive functioning and social cognition domains, thought to be more impacted in bvFTD and the PPAs (Rascovsky et al., 2011; Gorno-Tempini et al., 2011; Schroeter et al., 2018). Behavioral and psychiatric changes, as well as impairment in activities of daily living were assessed using questionnaires. All variables included in the analysis are summarized in Table 2. Descriptive statistics for the variables included in the clustering implementation are shown in Supplementary Tables S1, S2 for the analysis without and with questionnaires, respectively.

**Table 2:** List of variables included in the analysis. In brackets the range of test scores is specified where possible.

Category	Variables used for analysis (range)
Demographics	
	Age
	Years of education
Screening tools	
(FTLD-)CDR	FTLD-CDR sum of boxes (0–18)
	CDR sum of boxes (0–12)
	Memory (0–3)
	Orientation (0–3)
	Judgment and problem solving (0–3)
	Community affairs (0–3)
	Home and hobbies (0–3)
	Personal care (0–3)
	Behavior, comportment and personality (0–3)
	Language (0–3)
MMSE	CERAD MMSE total score (0–30)
Neurocognitive domain	
Complex attention	CERAD TMT A (0–180)
	Stroop task-color naming
	Stroop task-word reading
Perceptual-motor function	CERAD visuo-constructional praxis copy (0–11)
Language	CERAD Boston naming test (0–15)
	Repeat and point test–point (0–10)
	Repeat and point test–repeat (0–10)
	CERAD semantic fluency
	CERAD phonemic fluency
	Cookie theft task (0–20)
	AAT token test (0–50)
	AAT written language test (0–90)
Learning and memory	CERAD word list immediate recall 1 (0–10)
	CERAD word list immediate recall 2 (0–10)
	CERAD word list immediate recall 3 (0–10)
	CERAD word list immediate recall total (0–30)
	CERAD word list delayed recall (0–10)
	CERAD word list delayed savings
	CERAD word list recognition (0–100)
	WMS-R digit span forward (0–12)
	WMS-R visual memory span forward (0–14)
	CERAD visuo-constructional praxis delayed recall (0–11)
	CERAD visuo-constructional praxis delayed savings
Executive function	CERAD TMT B (0–300)
	CERAD TMT error scores
	CERAD TMT ratio B/A
	WMS-R digit span backward (0–12)
	WMS-R visual memory span backward (0–12)
	Stroop color-word interference
	Stroop error score
	H5PT total
	H5PT total correct
	H5PT percent correct (0–100)
	Cognitive estimation (0–16)
	Applause test (0–6)
Social cognition	RMET (0–24)
Questionnaires	
AES	AES total score (0–54)
B.ADL	B.ADL total score (1–10)
FrSBe	Executive dysfunction frequency (8–40)
	Disinhibition frequency (8–40)
	Apathy frequency (8–40)
GDS	GDS total score (0–15)

AAT, Aachen aphasia test; AES, apathy evaluation scale; B.ADL, Bayer activities of daily living; BNT, Boston naming test; CDR, clinical dementia rating; CERAD, consortium to establish a registry for Alzheimer's disease; FrSBe, Frontal Systems Behavior Scale; FTLD-CDR, frontotemporal lobar degeneration clinical dementia rating; GDS, geriatric depression scale; H5PT, Hamasch five point test; MMSE, mini-mental state examination; RMET, reading the mind in the eyes test; TMT, trail making test; WMS-R, Wechsler memory scale revised.

### 2.3 Statistical analysis

Statistical analysis was performed entirely using RStudio (RStudio Team, 2020) version 4.2.0. Analysis was restricted to the first visit of participants with a focus on earliest disease stages. Diagnostic groups were inspected on differences in demographic variables (Table 1). For the subsequent analysis, the two numerical demographic variables age and years of education were included. Each neuropsychological variable was checked for implausible values. Nine datapoints fell outside the defined test range of the respective variables and thus were coded as missing value.

#### 2.3.1 Multiple imputation

Multiple imputation was performed using the mice package (van Buuren and Groothuis-Oudshoorn, 2011) for multiple imputation by chained equations. In case both sub- and total scores were not present in the data, sub-scores were imputed, and total scores derived from the imputations *post hoc*. Imputation was performed 10 times and for a maximum of 100 iterations. To ensure that results of imputation would yield realistic values within the ranges of the variables used, paired mean matching was defined as imputation method. Convergence was ensured by inspecting mean and standard deviation across datasets and iterations as described by van Buuren (2019). Next, density distributions of the original and the imputed data were visually inspected.

#### 2.3.2 Clustering analysis

Prior to *k*-means clustering analysis, variables that were inversely scored were reversed for higher scores to consistently indicate better performance. In the next step the scores were rescaled to vary between −1 and 1 to avoid range effects across variables (e.g., Brigadoi et al., 2017). *K*-means clustering was implemented using the *kmeans* function from the *stats* package (Core Team R, 2021). The *k*-means algorithm defined by Hartigan and Wong (Hartigan and Wong, 1979) was used. The number of random initializations was set to nstart = 100 with a maximum number of iterations set to iter-max = 400. In this paper a dimensional approach was taken, in which diagnostic decisions on a spectrum consider external factors, such as available treatment options (Murley et al., 2020). Previous clustering studies have used an internal validation metric such as the elbow method [e.g., (Alexander et al., 2021; Hoffman et al., 2017)] to define the number of clusters best representing the data. In this paper internal validation was not calculated. Instead, analyses were systematically repeated with a varying number of clusters. This allowed for flexible exploration of the data focusing on consistent results across analyses (von Luxburg et al., 2012). Results were evaluated with an external validation metric.

Number of clusters was set to vary between *k* = 2 and *k* = 9. Groups included in the analyses were (a) all groups, (b) only the patient groups, (c) bvFTD and PPAs, (d) only PPAs and (e) bvFTD and amnestic AD. Analyses were performed on each one of the 10 imputed datasets before combining the results using majority voting. Quality checks and their results are detailed in the Supplementary materials. For external validation, correspondence of clustering results with the pre-existing diagnostic labels was calculated using adjusted mutual information (AMI). Due to the unsupervised nature of *k*-means clustering the diagnostic labels had not been included in the clustering analysis, thus serving as reference clinical diagnosis for external validation. AMI evaluates results based on homogeneity and completeness while correcting for chance level [e.g., (Ficiarà et al., 2021)]. Clustering results were further evaluated visually on whether clusters separated between different diagnostic groups and how this changed with increasing number of clusters. Next, results were inspected more in detail to find neuropsychological profiles explaining the clustering observed.

Based on the exploratory nature of the clustering analysis, results will be summarized in a descriptive way as was performed in previous studies [e.g., (Ficiarà et al., 2021; Maruta et al., 2015)]. Focus is on consistent findings observed across cluster sizes and comparisons.

## 3 Results

### 3.1 Consensus clustering

#### 3.1.1 Varying the number of clusters and diagnostic groups in the analysis

By varying both the patient groups included and the number of clusters defined in the analysis (see section 2.3.2, second paragraph), we observed that increasing the number of clusters has an effect of “zooming in” to the data structure. Similar results were obtained when including fewer groups or by increasing the number of clusters. Analysis allowed us to observe that some groups separate more easily than others. A two-dimensional representation of the data based on Principal Component Analysis (PCA) can be found in the Supplementary Figure S6.

#### 3.1.2 Cluster results

**Figure 2:**
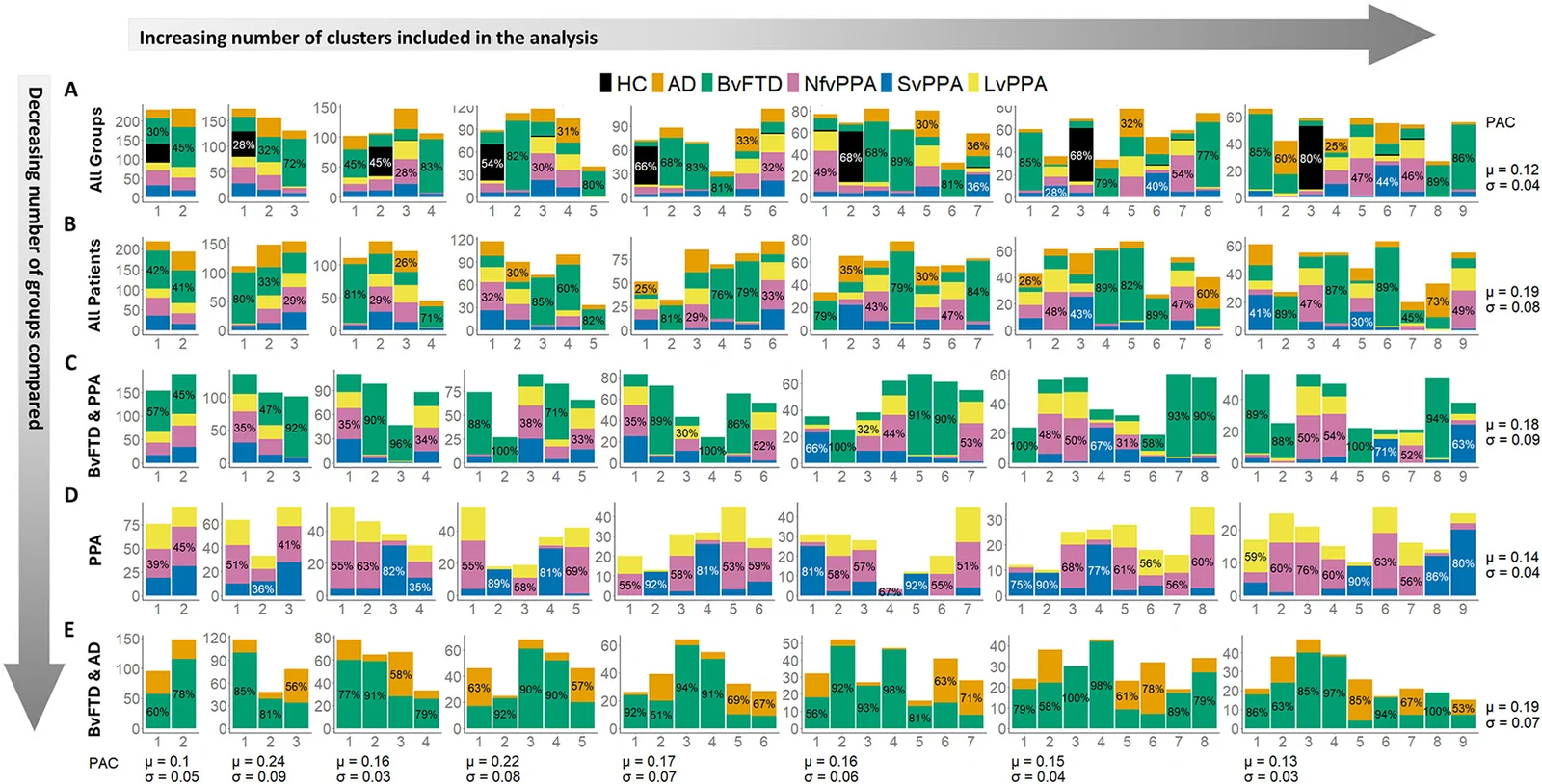
Composition of clusters by participant group in the analysis (including questionnaires). Analysis was repeated for cluster sizes 2–9 (left to right). Additionally, the analysis was repeated including specified participant groups **(A)** all groups, **(B)** all patient groups, **(C)** bvFTD and PPA groups, **(D)** PPA groups, **(E)** bvFTD and amnestic AD. Percentages displayed in the plot correspond to within-cluster proportions and were annotated for the largest group within a cluster. Mean and standard deviation of the proportion of ambiguous clustering (PAC) are shown for the group comparisons (A to E) and the number of clusters (*k* = 2–9) on the right and bottom of the plot, respectively. AD, amnestic Alzheimer's disease; bvFTD, behavioral variant frontotemporal dementia; lvPPA, logopenic variant primary progressive aphasia; nfvPPA, non-fluent variant primary progressive aphasia; PAC, proportion of ambiguous clustering; svPPA, semantic variant primary progressive aphasia.

To evaluate the results from the cluster analysis, the distribution of diagnostic groups within each cluster was visualized and is shown in Figure 2 for the analysis including behavioral questionnaire scores. The corresponding results from the analysis without questionnaires are shown in Supplementary Figure S5. Overall, results were considerably stable across various analyses. While no cluster showed complete correspondence with one diagnostic group, coherence between patient's diagnosis and the cluster results was partly supported. This was particularly true for participants diagnosed with bvFTD or svPPA, who, to a great proportion clustered together and separately from other diagnostic groups. Healthy controls clustered together but not separately from patients. By increasing *k*, healthy controls progressively separated better from patient groups reaching maximum homogeneity at *k* = 9 (excluding questionnaires: 75%, including questionnaires: 80%). Participants diagnosed with amnestic AD did not separate well from other diagnostic groups. Amnestic AD separated well from healthy controls while mixing to a similar extent with all other patient groups. In the analysis including only bvFTD and amnestic AD, maximum homogeneity reached by an amnestic AD cluster was 74% and 85% excluding and including questionnaire scores, respectively. Further, nfvPPA and lvPPA consistently clustered together while clustering apart from other groups. At low number of clusters, the three PPAs grouped together but separately from other groups. At a higher number of clusters, single clusters emerged that consisted mainly of svPPA or a combination of nfvPPA and lvPPA.

**Figure 3:**
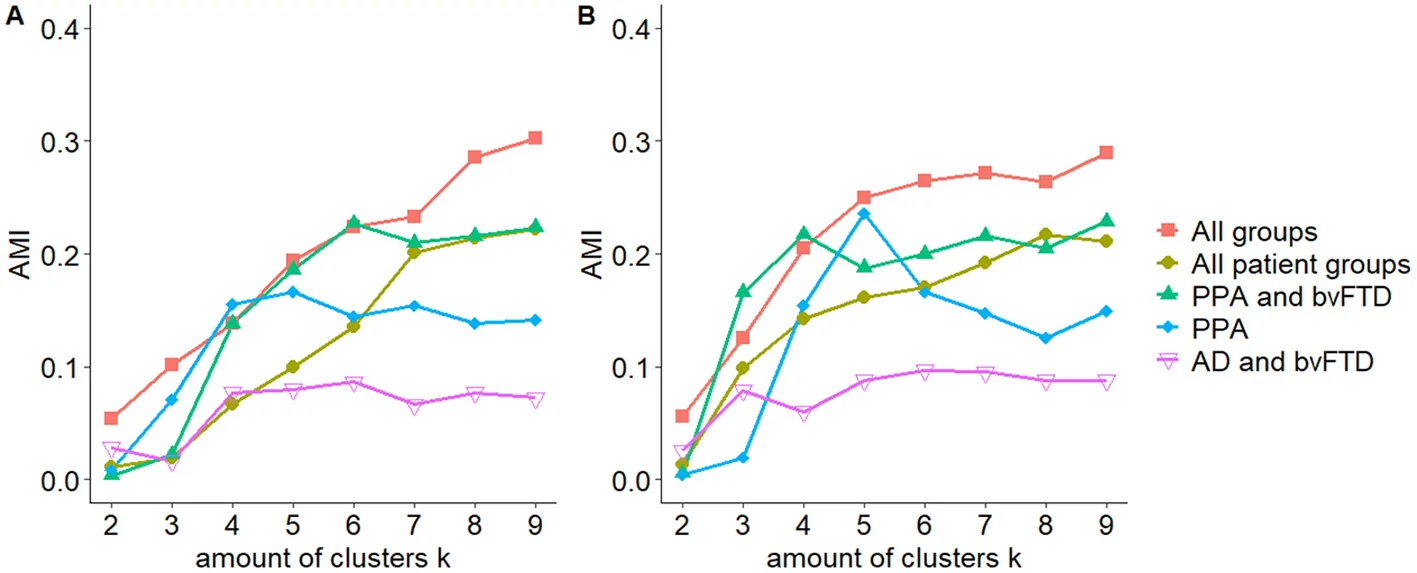
Adjusted mutual information (AMI) was calculated to quantitatively evaluate correspondence of clustering results with attributed diagnostic groups. **(A)** AMI in the analysis excluding questionnaires. **(B)** AMI in the analysis including questionnaires. Generally, by increasing number of diagnostic groups included, maximum AMI was reached with higher number of clusters. Comparing graphs A (without questionnaires) and B (with questionnaires) may demonstrate particular importance of questionnaires in discriminating patients with bvFTD. In the analyses including bvFTD a maximum AMI was reached with a lower number of clusters. AD, amnestic Alzheimer's disease; AMI, adjusted mutual information; bvFTD, behavioral variant frontotemporal dementia; PPA, primary progressive aphasia.

External evaluation using AMI is shown in Figure 3. This score varies between zero and one and indicates correspondence of cluster results with the diagnosis registered in the data set. By increasing the number of groups included in the analyses AMI leveled at a higher number of clusters: leveling off at 2–3 clusters when including amnestic AD and bvFTD, 4–5 clusters when including PPA and 4–6 clusters when including PPA and bvFTD. When including more groups (all patients or all groups), no clear leveling off was observed, possibly suggesting that more clusters would be needed to capture possible groups in the data.

### 3.2 Inclusion of questionnaire scores

**Figure 4:**
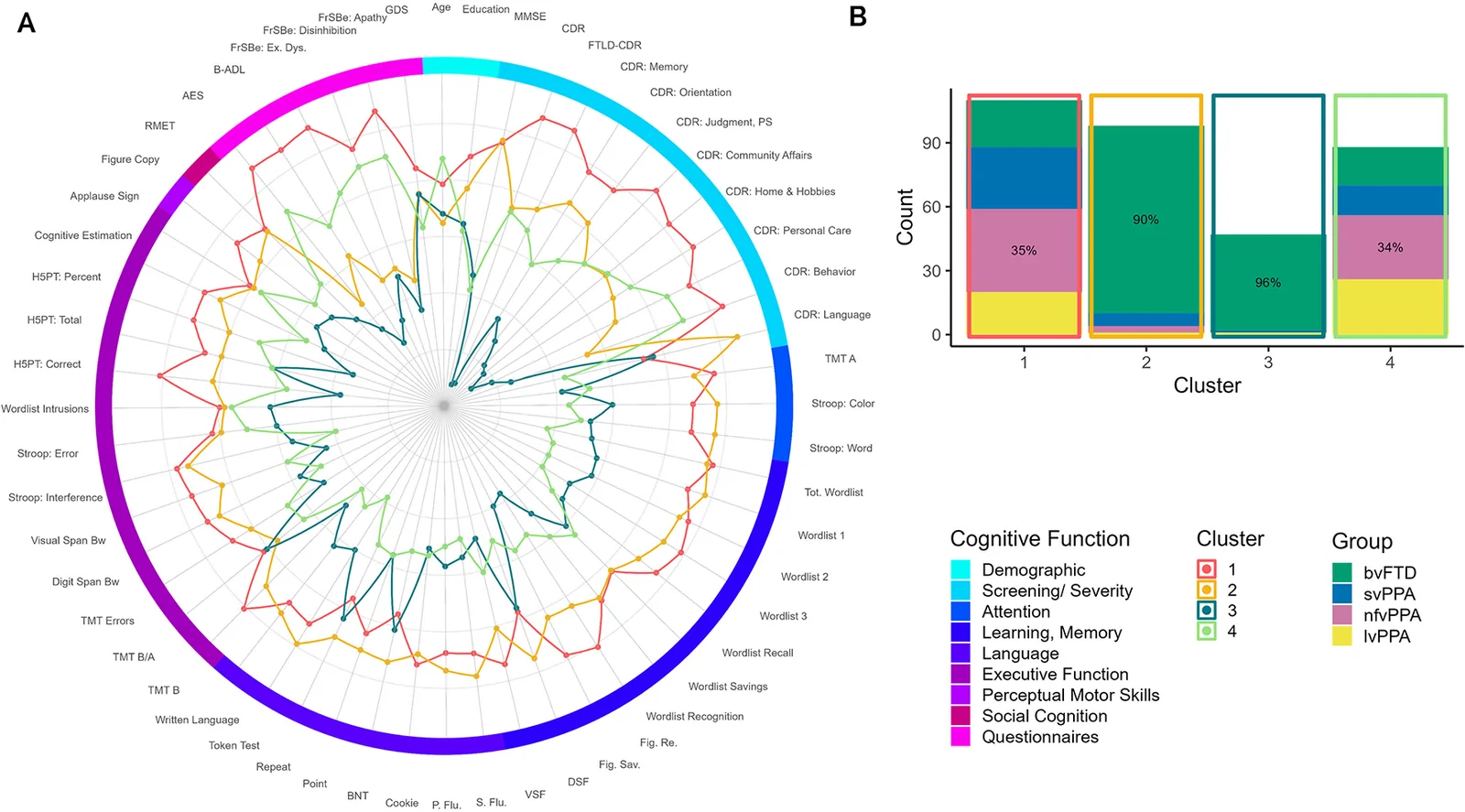
Neuropsychological profiles of cluster results (including questionnaires, analysis including PPA and bvFTD, *k* = 4). Example plot representing neuropsychological profiles for the analysis including patients with PPA and bvFTD, including questionnaires and limiting the analysis to *k* = 4 clusters. For the analysis scores were scaled to vary between −1 and 1. For visualization purposes only, cluster centers were z-standardized. Scores more at the inside of the radar plot correspond to worse performance. Comparing clusters 2 (yellow) and 3 (dark green) that both consist mainly of bvFTD patients, cluster 2 performs better on virtually all assessments indicating an influence of symptom severity on clustering results. Compared to clusters 1 (red) and 4 (light green) these two clusters perform particularly bad on the questionnaires, the FTLD-CDR subscores “Home and Hobbies”, “Personal Care” and “Behavior, comportment and personality”. **(A)** Radar Plot indicating z-standardized cluster centers for clusters 1–4. **(B)** Box plot indicating patient groups within clusters 1–4. Percentages displayed in the plot correspond to within-cluster proportions and were annotated for the largest group within a cluster. AES, Apathy Evaluation Scale; B-ADL, Bayer activities of daily living; BNT, Boston naming test; BvFTD, behavioral variant frontotemporal dementia; CDR, Clinical Dementia Rating Scale; DSF, digit span forward; Ex. Dys, executive dysfunction; Fig. Re, figure recall (from CERAD battery); Fig. Sav, Figure savings (from CERAD battery); FrSBe, Frontal Systems Behavior Scale; FTLD-CDR, Frontotemporal Lobar Degeneration Clinical Dementia Rating Scale; GDS, Geriatric Depression Scale; H5PT, Hamasch five point Test; LvPPA, logopenic variant primary progressive aphasia; MMSE, mini-mental state examination; NfvPPA, non-fluent variant primary progressive aphasia; TMT, trail making test; P. Flu, phonematic fluency, RMET, reading the mind in the eyes test; S. Flu, semantic fluency; SvPPA, semantic variant primary progressive aphasia; VSF, visual span forward.

Systematically varying the inclusion of questionnaire scores in the analysis showed that scores of companion-rated questionnaires focused on behavior helped in distinguishing bvFTD from other groups, particularly from PPAs and healthy controls (Figure 2 and Supplementary Figure S5). Including questionnaires enabled a separation of bvFTD from other groups at lower number of clusters. When including PPA and bvFTD, inclusion of questionnaires seemed to favor separation of bvFTD while making separation of PPA groups more difficult, requiring larger number of clusters for svPPA to cluster apart. In the analysis with all groups, excluding questionnaires resulted in a greater proportion of bvFTD patients clustering with healthy controls also at a high number of clusters (compare Figure 2 and Supplementary Figure S5 part A at *k* = 6 and *k* = 7). Questionnaire scores that seemed of special relevance were the Frontal Systems Behavior Scale (FrSBe) and the Apathy Evaluation Scale (AES; e.g., see Figure 4). The usefulness of these questionnaires for differential diagnoses between non-bvFTD groups seemed limited as expected.

### 3.3 Neuropsychological profile

Next, radar plots visualizing scores of clusters across the range of neuropsychological tests, were inspected. Large heterogeneity was observed both within and between diagnostic groups and explains the fuzzy separation of diagnostic groups observed previously in Figure 2 (also, see Supplementary Figure S5). The large majority of healthy controls grouped in a single cluster which corresponded to the best performing cluster on virtually all tests. Interestingly, the cluster containing healthy control participants was partly outperformed on the FrSBe Disinhibition scale by clusters containing most nfvPPA and lvPPA patients. Further, one consistent finding was a binary influence of symptom severity on clustering results. At low number of clusters (*k* = 2 and *k* = 3), symptom severity seemed to be driving the cluster separation. When several clusters emerged that mainly consisted of the same diagnostic group, the difference between clusters seemed in many cases attributable to symptom severity (see as example clusters 2 and 3 in Figure 4). At higher number of clusters, disease-specific patterns were observed. In the following, consistent disease-specific patterns are described for the FTLD subgroups. Exemplary figures illustrating the findings are shown below (Figure 4) and in the Supplementary Figures S7–S15).

#### 3.3.1 BvFTD

Clusters with mainly bvFTD patients scored worse than other clusters on FTLD-CDR subscores, particularly on “Behavior, comportment, & personality”, “personal care”, “Home & hobbies” and “personal affairs” (Figure 4). Further, a considerable difference between the “Language” and the “Behavior, comportment, & personality” subscores of the FTLD-CDR was observed in comparison to PPA clusters. While bvFTD scored considerably better on the “Language” subscore, the opposite was the case for PPA clusters. Additionally, lower scores on companion-rated questionnaires, especially on AES and FrSBe, separated bvFTD from other participant groups. This was the case also when cognitive performance was similar to healthy controls. In contrast, at high levels of cognitive impairment, bvFTD clusters maintained good performance on the BNT and repeat and point task.

Separation of amnestic AD and bvFTD in different clusters was incomplete, complicating interpretation of results. Particularly scores on executive and memory assessments did not seem to differ meaningfully between these two groups. However, amnestic AD clusters mirrored performance of the most impaired bvFTD clusters on memory scores, suggesting greater relative rather than absolute impairment on these tests.

#### 3.3.2 PPA

Generally, PPA clusters showed a great drop from the “Behavior, comportment and personality” subscore to the “Language” subscore. One cluster that in many cases consisted mainly of lvPPA and nfvPPA patients, showed lowest performance on the MMSE while the cluster performing worst on the FTLD-CDR usually consisted mainly of bvFTD patients. SvPPA clusters performed worst on BNT and the point part of the repeat and point task compared to other clusters.

Comparing svPPA and nfvPPA/lvPPA clusters, a different pattern emerged on the repeat and point task for clusters containing svPPA patients compared to clusters containing patients diagnosed with lvPPA and nfvPPA (e.g., see Supplementary Figure S12). While absolute scores varied largely in both types of clusters, lvPPA and nfvPPA patients scored similarly on both repeat and point while svPPA patients showed better performance on the repeat compared to the point part of the task. Additionally, compared to lvPPA and nfvPPA, svPPA clusters scored lower on the FTLD-CDR subscore “Behavior, comportment, and personality”. Generally, patients with svPPA scored low on BNT, Cookie Theft and verbal but not phonemic fluency assessments. LvPPA and nfvPPA patients instead showed greater impairment on tasks with smaller semantic but greater attention and executive components. For example, these clusters typically scored worse on the TMT A and B, digit span, block span and the Stroop, compared to svPPA clusters. This may indicate that these patients have greater problems in tasks assessing non-language domains. However, despite these tasks being developed to assess non-language domains, particularly the TMT B, digit span and Stroop, rely on language processing and speech production. Thus, one may question whether deficits observed in these tasks are a side-effect of underlying problems with language processing. One interesting question for future investigations will be whether these tasks could be more sensitive to differential diagnosis between svPPA and nfvPPA/lvPPA.

## 4 Discussion

The present study used an unsupervised ML algorithm to analyze neuropsychological and behavioral data from patients with FTLD. Comparison groups included in the study were healthy controls and patients with amnestic AD and associated lvPPA, to contain the clinically most relevant groups. The goal of the study was to explore neuropsychological profiles from these different groups and compare results with the current clinical diagnostic criteria. Differential diagnosis of these patient groups remains difficult, and misdiagnoses are common (Rascovsky and Grossman, 2013). To date, diagnostic certainty can be reached only via pathological confirmation post-mortem (Deuschl et al., 2016) and there is no gold-standard for diagnosis prior to death. Currently, biomarker profiles from imaging, cerebrospinal fluid and blood are being developed (Arevalo-Rodriguez et al., 2021; Hüper et al., 2024).

To summarize results, meaningful correspondence between patient's diagnosis and clustering results was observed across repeated analyses of varying patient groups, variables (including or excluding questionnaires), and numbers of clusters *k* defined for the analysis. Segregation in individual clusters was more pronounced for bvFTD and svPPA supporting the assumption of specific clinical profiles. Interestingly, amnestic AD patients mixed with all other patient groups to a similar extent. NfvPPA and lvPPA patients–both representing non-fluent PPA subtypes in contrast to fluent svPPA–grouped together but separately from the other groups. Remarkably, these results correspond to imaging studies applying ML to classify FTLD subtypes based on their different atrophy patterns (Lampe et al., 2022, 2023). While svPPA could be identified with high accuracy, the separation between lvPPA and nfvPPA was impossible, operating almost at chance level (Bisenius et al., 2017). This underlines the importance of fluid biomarkers for the differentiation of nfvPPA and lvPPA (Hüper et al., 2024). Alternatively, the used tests assessing cognitive and particularly language functioning may not be sufficiently sensitive for the distinction of lvPPA and nfvPPA. Particularly, no tasks assessing phonological working memory or speech apraxia were included in the current study.

Further, the findings of the current study supported the following aspects that are in accordance with former studies and disease concepts: (1) A hierarchical procedure of diagnosing PPA before defining the exact PPA variant is in accordance with current diagnostic criteria (Gorno-Tempini et al., 2011). A particularly high score on the Language item of the FTLD-CDR, indicating high levels of impairment, may be sensitive to PPA. A general instrument such as the FTLD-CDR, to a large extent based on informants' observations, may thus be used in a first step to understand impairment of language functioning above other cognitive/functional domains. In a second step, formal, quantitative neuropsychological testing of language deficits, particularly of semantic processing via the BNT and the Repeat and Point, may instead be useful and required to distinguish svPPA from nfvPPA/lvPPA. A drop of performance from the repeat to the point task as indicative of svPPA symptomatology was supported by the current study (Henry and Grasso, 2018; Seckin et al., 2022). Apart from language assessments, results highlight the possible added value of including assessments of other cognitive domains, particularly of attention and executive function in the differential diagnosis of PPA variant. (2) In accordance with current diagnostic criteria, separation of bvFTD from other diagnostic groups seemed to be facilitated by including measures of changes in behavior rather than cognition. So far, the best way to detect these changes are companion-rated questionnaires, e.g., AES and FrSBe and the FTLD-CDR Behavior, comportment, & personality (Rascovsky et al., 2011). (3) Memory impairments in bvFTD may be as pronounced as in amnestic AD. Sparing of memory dysfunction as stated in the diagnostic criteria of bvFTD is only true relative to other dysfunction (Rascovsky et al., 2011). A dissociation of amnestic AD and bvFTD may only be observed when evaluating memory relative to general cognitive functioning. This was shown in the current study using the (FTLD-) CDR. BvFTD patients showed either relatively preserved memory as well as (FTLD-) CDR global scores or high impairment on both dimensions. Amnestic AD instead showed greater relative impairment on memory functions compared to their FTLD-CDR scores, serving as measure of disease severity.

Furthermore, the current study revealed findings contradicting former literature. While bvFTD was related to worse scores on the FrSBe Executive Dysfunction scale, standard assessments of executive functioning may not aid separation of bvFTD from the other patient groups. A review by Johnen and Bertoux (2019) previously highlighted the usefulness of the TMT B error score which did however not show meaningful distinction of bvFTD and other groups in the current study. Similarly, despite previous studies suggesting social cognition to differentiate bvFTD from healthy controls (Schroeter et al., 2018) and other neurodegenerative or psychiatric diagnoses (Gossink et al., 2018; Johnen and Bertoux, 2019; Ducharme et al., 2020) the current study did not find supporting evidence. Additional results suggest that cognitive involvement in the PPA variants may be larger than might have been expected and may exceed the language domain. This was found despite data in the current study being restricted to patient's first visit and average delay since symptom onset for PPA patients being less than 3 years. SvPPA and nfvPPA/lvPPA clusters differed on assessments of processing speed, short term and working memory. Previous studies indicated that patients with lvPPA show greater cognitive deficits compared to both nfvPPA and svPPA (Kamath et al., 2020). Additionally, impairment in working memory tasks was shown in both lvPPA and nfvPPA (Harris et al., 2019) while patients with svPPA may not be impaired in these tasks (Foxe et al., 2021; Kamath et al., 2020). Distinction of lvPPA from nfvPPA based on verbal and visuospatial working memory tasks could however not be replicated.

The findings of this study highlight the heterogeneity of neuropsychological profiles within diagnostic groups, as well as considerable overlap between them. This contrasts with clinical diagnostic criteria suggesting relatively circumscribed impairments in episodic memory, language or executive functioning for amnestic AD (McKhann et al., 2011), the PPAs (Gorno-Tempini et al., 2011) and bvFTD (Rascovsky et al., 2011), respectively. Importantly, our results suggest that the variability observed may largely be explained by differences in disease severity. Greater overlap in clinical symptoms between patient groups may be explained by expansion of neuropathology (Bürger et al., 2017). Consequently, differences in disease severity may partly obscure disease-specific cognitive profiles.

These findings highlight the importance of comprehensive neuropsychological examination across all six cognitive domains described in the DSM-5 (Sachdev et al., 2014) to interpret results from one cognitive domain with respect to results in other domains. Additionally, neuropsychological testing may benefit from interpretation relative to disease stage. While z-standardized scores remain essential for characterizing impairment compared to healthy controls, as well as for treatment planning, differential diagnosis may benefit from stage-specific comparisons. Future studies may therefore benefit from focusing on clearly defined disease stages, whether by stratifying patient groups based on staging instruments such as the FTLD-CDR or by applying ML tools such as subtype and stage inference (SuStaIn) (Young et al., 2018). Lastly, longitudinal follow-up of patients may provide insights into expected changes of cognitive profiles with disease progression and improve interpretation of neuropsychological results in the context of differential diagnosis.

### 4.1 Limitations and future studies

We did not find evidence suggesting that social cognition or executive function assessments aid the diagnosis of bvFTD. This may be linked to the tests used in the current study. For social cognition a single assessment, with high demands on semantic processing and a considerable proportion of missingness was included. Possible differences between participants on this task may have been washed out by algorithmic limitations and noise. Additionally, both executive and social function are complex constructs and relevant impairments may not have been reflected appropriately by the assessments used. The results encourage the investigation of alternative tests that minimize language barriers. Tests previously suggested for differential diagnosis include the Social Cognition and Emotional Assessment (SEA) (Bertoux et al., 2012), the ultimatum game (Hinterbuchinger et al., 2018), or tasks developed for the observation of spontaneous social behavior (Rankin et al., 2008). Concerning executive function, more recent studies suggest that the inclusion of a summed executive error index, calculated from perseverations, rule violations and stimulus-bound errors, may help to detect executive problems in bvFTD (Kamath et al., 2019; Kramer et al., 2003). Furthermore, executive function tests such as the Behavioral Assessment of the Dysexecutive Syndrome (BADS) might be applied that tap more in everyday life functions than classical tests (Schroeter et al., 2012). Future work is necessary to investigate sensitive and specific neuropsychological tests for bvFTD and diagnostic criteria need to be refined accordingly.

Biological sex was not included as demographic variable in the current analysis while recent studies suggest that sex has an impact on both clinical symptoms and extent of brain atrophy in dementia syndromes. Although results are not conclusive and may be limited if sex (and, in particular, gender) is regarded as binary, sexual dimorphism was shown for PPA (Rogalski et al., 2007; Sebastian et al., 2018), bvFTD (Illán-Gala et al., 2021) and AD (Gamberger et al., 2016).

Furthermore, one might criticize that surrogate biomarkers for histopathology from imaging, cerebrospinal fluid and blood were not included. These are particularly relevant for the identification of Alzheimer's pathology in amnestic AD and lvPPA (Hüper et al., 2024). However, our paper focused on behavioral and cognitive measures, and the results of these biomarkers were included in the diagnostic procedure to define diseases in our cohort (Hüper et al., 2024). Results from this clinical “gold-standard” were used as external validation of the clustering results.

In contrast to previous clustering studies (Cerami et al., 2016; Owens et al., 2018) the current study did not support the existence of robust subgroups within current diagnoses. However, diagnostic precision may always present a trade-off between simplifying the clinical picture of a patient while remaining meaningful for treatment or disease management. Instead of aiming to find the number of subgroups best explaining the data, as performed in previous studies using internal validation metrics, we intended to explore data structure flexibly. By evaluating results that we observed repeatedly across analyses, results may be independent of algorithmic choices, such as the number of clusters or the groups included for comparison and thus allow for generalization to other analyses. *K*-means clustering seemed very suitable for multiclass analysis including patient groups from various etiologies. Despite high clinical relevance, multiclass analysis is not common in unsupervised learning and to our knowledge the current study was the first one to jointly investigate amnestic AD, bvFTD and (the three subtypes of) PPA in this way. We propose to use these methods for exploration of patterns, heterogeneity, and overlap. Nonetheless, exploration remains limited by the method of clustering used and the means of data visualization at hand (von Luxburg et al., 2012). *K*-means clustering used in the current study is one of several clustering methods, each method relying on specific assumptions. *K*-means clustering aims to minimize clustering error, expressed as sum of the squared Euclidean distance, thereby favoring spherical clusters with similar cluster sizes, also referred to as the “uniform effect” (Zhou and Yang, 2020). Applying alternative clustering approaches may lead to slightly different results and may shed light on new aspects of the disease spectrum.

Lastly, some clusters emerged that remained mixed and were difficult to interpret. Missing pathological or neuroimaging information does not explain well the clusters that contained patients from all or most diagnostic groups. It may demonstrate the heterogeneity of cognitive symptoms observed for patients of the same diagnostic group. Additionally, it is conceivable that these clusters contain misdiagnoses. Pathological data existed only for a minority of participants included in the study. Future studies may investigate these mixed clusters more closely to understand whether additional, multimodal data could help the distinction of these groups.

## 5 Conclusion

Using a data-driven approach on a breadth of neuropsychological and behavioral data, this study demonstrated substantial heterogeneity in clinical profiles within diagnostic groups across the FTLD and AD spectrum, as well as overlap between them. Despite focusing on the baseline visit, heterogeneity within and overlap between groups could partly be explained by differences in disease severity. At higher number of clusters, patients with svPPA and bvFTD clustered separately from other patient groups, while grouping of the remaining patient groups included was more mixed. LvPPA and nfvPPA–as non-fluent PPA variants–did not separate well based on the cognitive and behavioral data included in the study.

Focusing on clusters with similar disease severity, similar syndrome-specific patterns were observed across analyses. High relevance of companion-rated questionnaires assessing apathy, executive dysfunction and disinhibition was shown for separating patients with bvFTD from other groups. Semantic impairment in svPPA was shown by low scores on both language and memory tests. NfvPPA and lvPPA patients instead showed impairment in processing speed and short term and working memory assessments. Results from this study support the need for revising the neuropsychological profile included in the clinical diagnostic criteria of bvFTD. Differential diagnosis of PPA variants may instead benefit from including additionally specifications of expected impairments on assessments that do not primarily focus on language functioning.

In conclusion, these findings–partial homogeneous clustering and syndrome-specific profiles–underscore the relevance of a patient-centered understanding of patients' neuropsychological and behavioral profiles. Data-driven approaches that are independent of prior labeling of the data, may therefore prove particularly useful for analyzing data from highly heterogeneous diagnostic groups or unclassifiable patients.

## Data Availability

The datasets presented in this article are not readily available because it contains sensible patient information. Data in anonymized form will be made available on reasonable request. Requests to access the datasets should be directed to Marie.Soentgerath@uk-halle.de.
