# Supplementary material for: Evaluating the correspondence of neuropsychological profiles with clinical diagnostic criteria for frontotemporal lobar degeneration and Alzheimer's disease: insights from k-means clustering
Source: Front Dement. 2026 Aug 31;5:1757680. doi: 10.3389/frdem.2026.1757680 (PMC13613979; doi:10.3389/frdem.2026.1757680)
Supplement: Supplementary file 1 [file Data_Sheet_1.pdf]

Supplementary Material

List of Neuropsychological Tests and Questionnaires

- Clinical Dementia Rating (CDR) (1) and FTLD-CDR Scales (2)
- CERAD-NAB Plus Battery (3,4): semantic fluency (5), phonological fluency (6), modified Boston Naming Test (BNT) (7), Minimental State Examination (MMSE) (8), verbal memory test (9,10), constructional praxis (11), Trail Making Test (TMT) (12)
- Modified Stroop Task (13)
- Wechsler Memory Scale Revised (WMS-R) (14)
- Cookie Theft Task (15)
- Repeat and Point Test German Adaptation (16)
- Aachen Aphasia Test (AAT) (17): Token Test, Written Language Test
- Hamasch Five Point Test (H5PT) (18)
- Cognitive Estimation Task (19) updated version (20)
- Reading the Mind in the Eyes Test (RMET) Revised Version (21)
- Three Clap Test (TCT) (22)
- Apathy Evaluation Scale (AES) (23): patient and informant versions
- Bayer Activities of Daily Living Scale (B.ADL) (24): patient and informant versions
- Frontal Systems Behavior Scale (FrSBe) (25): patient and informant versions
- Short Form Geriatric Depression Scale (GDS) (26)

Missing Data

Due to high levels of missing data, several steps were taken to prepare the data for the k-means clustering analysis. Exploration of the pattern of missingness across variables and diagnostic groups indicated that patients had particularly high levels of missingness in the self-rating parts of the three questionnaires (*i.e.,* Apathy Scale, BADL, FrSBe) while healthy controls had particularly high levels of missingness in the informant-rating parts of the same questionnaires (Figure S1). To accommodate for this observation, we decided to minimize missingness by including only the informant-based scores for the patient groups while including only the self-rating scores for healthy controls. This decision was supported by the observation from previous studies that informant-based questionnaires may be more indicative of behavioral changes of the patients than self-ratings [e.g., (27)]. In the case of control participants, no behavioral abnormalities are expected and thus informant- and self-ratings are not thought to differ meaningfully. Importantly, this process was equivalent in all patient groups. We opted for this procedure rather than simply excluding questionnaire scores, as the information contained in the questionnaires might have an added value, especially in the differential diagnosis of bvFTD, whose clinical picture is thought to be characterized primarily by behavioral rather than cognitive changes. Additionally, missingness observed in the current dataset might reflect difficulties collecting questionnaire data in the clinical setting. To ensure that results would not be obscured by large proportion of imputed data, the analysis was performed twice - once including and once excluding questionnaire scores – allowing us to investigate robustness of results and to estimate the usefulness of questionnaire data for differential diagnosis.

Next, list-wise deletion of those participants who exceeded the threshold of 20% missingness in the variables relevant for analysis was performed. In accordance with the current standard (28–30), missing data in the remaining participants was imputed using MICE (31). In cases

where missingness is not completely at random and proportion of missing data exceeds few percent points (usually a threshold of 5-10%), a listwise deletion may severely bias the results and may not be representative for the larger sample or population (32). In these cases, multiple imputations are considered a powerful way to deal with missing data (33). In comparison to single imputation, multiple imputations do not replace each missing value with a single but with multiple, commonly five or more, values. This creates several datasets on which analysis is then performed before results are combined in a last step. Compared to single imputation, this procedure avoids underestimating standard errors. As analysis is performed on each imputed dataset, one can inspect how much the results from the repeated analyses diverge. Results that vary highly across imputations should be interpreted with care.

Robustness of Results

Cramer's V

Data was imputed 10 times creating ten datasets on which the analysis was performed. Results from the 10 datasets were inspected on similarity using Cramer's V. Similarly to a correlation index, this measure assesses correspondence of the clusters attributed to participants across the analyses performed on the ten imputed datasets. In the majority of analyses Cramer's V varied between 0.7 and 1 for pairs of datasets (Figure S3, Table S4, S5). It dropped below 0.7 for single datasets only. Generally, Cramer's V decreased with increasing number of clusters but remained high even for the maximum number of clusters.

Proportion of Ambiguous Clustering

Before combining results using majority voting, stability of clustering across the ten analyses was assessed using proportion of ambiguous clustering (PAC) (34). PAC gives the proportion of participant pairs, for which less than nine out of the ten analyses agree that the two participants should cluster together or separately. Lower bound of consensus indices were set to 0.1 and upper bound to 0.9. The results supported relative stability of clustering across analyses (Figure S4, Table S6, S7). In most cases, PAC ranged from 0.06 to 0.26, meaning that in more than two thirds of participant pairs at least nine out of ten datasets agreed that participant pairs should cluster together or separately. Analysis with PPA and bvFTD resulted in rather bad results on this index ranging from 0.13 to 0.37. With three clusters a PAC of 0.37 indicated that nearly 40% of participant pairs can be considered as ambiguously clustered. Except for this specific case, we concluded that clustering results across imputations were sufficiently stable for further analysis.

Next, consensus clustering was performed using the majority voting function from the diceR package (35). This method evaluates correspondence of cluster labels across different runs of the analysis before assigning to each participant the cluster that it was most often attributed to.

1. Morris JC. The Clinical Dementia Rating (CDR) Current version and scoring rules. Neurology. (1993) 43:2412.2-2412-a. doi: 10.1212/WNL.43.11.2412-a.

2. Knopman DS, Kramer JH, Boeve BF, Caselli RJ, Graff-Radford NR, Mendez MF, et al. Development of methodology for conducting clinical trials in frontotemporal lobar degeneration. Brain. (2008) 131:2957–68. doi: 10.1093/brain/awn234.

3. Moms JC, Heyman A, Mohs RC, Hughes JP, van Belle G, Fillenbaum G, et al. The Consortium to Establish a Registry for Alzheimer's Disease (CERAD). Part I. Clinical and neuropsychological assesment of Alzheimer's disease. Neurology. (1989) 39:1159–1159. doi: 10.1212/WNL.39.9.1159.

4. Fillenbaum GG, Belle G Van, Morris JC, Mohs RC, Mirra SS, Davis PC, et al. Consortium to Establish a Registry for Alzheimer's Disease (CERAD): The first twenty years. Alzheimer's & Dementia. (2008) 4:96–109. doi: 10.1016/j.jalz.2007.08.005.

5. Isaacs B, Kennie AT. The Set Test as an Aid to the Detection of Dementia in Old People. British Journal of Psychiatry. (1973) 123:467–70. doi: 10.1192/bjp.123.4.467.

6. Thurstone LL. Primary Mental Abilities. The Measurement of Intelligence, Dordrecht: Springer Netherlands; (1973), p. 131–6. doi: 10.1007/978-94-011-6129-9_8.

7. Kaplan E, Goodglass H, Weintraub S. The Boston Naming Test. Veterans Administration Medical Center. (1978).

8. Folstein MF, Folstein SE, McHugh PR. "Mini-mental state". A practical method for grading the cognitive state of patients for the clinician. J Psychiatr Res. (1975) 12:189–98. doi: 10.1016/0022-3956(75)90026-6.

9. Atkinson RC, Shiffrin RM. The Control of Short-Term Memory. Sci Am. (1971) 225:82–91.

10. Mohs RC, Kim Y, Johns CA, Dunn DD, Davis KL. Assessing changes in Alzheimer's disease: Memory and language. Handbook for clinical memory assessment of older adults., Washington: American Psychological Association; (1986), p. 149–55. doi: 10.1037/10057-012.

11. Rosen WG, Mohs RC, Davis KL. A new rating scale for Alzheimer's disease. American Journal of Psychiatry. (1984) 141:1356–64. doi: 10.1176/ajp.141.11.1356.

12. Reitan RM. Validity of the Trail Making Test as an Indicator of Organic Brain Damage. Percept Mot Skills. (1958) 8:271–6. doi: 10.2466/pms.1958.8.3.271.

13. Stroop JR. Studies of interference in serial verbal reactions. J Exp Psychol. (1935) 18:643–62. doi: 10.1037/h0054651.

14. Wechsler DA. Manual for the Wechsler Memory Scale-Revised. New York: Psychological Corporation. (1987).

15. Goodglass H, Kaplan E. The assessment of aphasia and related disorders. Lea & Febiger. (1972).

16. Heitkamp N, Leiss E, Danek A. Repeat & Point German Adaptation of a tool for differentiating semantic dementia and primary progressive aphasia. Klinische Neurophysiologie. (2010) 41.

17. Willmes K, Poeck K, Weniger D, Huber W. Facet theory applied to the construction and validation of the Aachen Aphasia Test. Brain Lang. (1983) 18:259–76. doi: 10.1016/0093-934X(83)90020-2.

18. Regard M, Strauss E, Knapp P. Children's production on verbal and non-verbal fluency tasks. Percept Mot Skills. (1982) 55:839–44. doi: 10.2466/pms.1982.55.3.839.

19. Shallice T, Evans ME. The Involvement of the Frontal Lobes in Cognitive Estimation. Cortex. (1978) 14:294–303. doi: 10.1016/S0010-9452(78)80055-0.

20. MacPherson SE, Wagner GP, Murphy P, Bozzali M, Cipolotti L, Shallice T. Bringing the Cognitive Estimation Task into the 21st Century: Normative Data on Two New Parallel Forms. PLoS One. (2014) 9:e92554. doi: 10.1371/journal.pone.0092554.

21. Baron-Cohen S, Wheelwright S, Hill J, Raste Y, Plumb I. The "Reading the Mind in the Eyes" Test Revised Version: A Study with Normal Adults, and Adults with Asperger Syndrome or High-functioning Autism. Journal of Child Psychology and Psychiatry. (2001) 42:S0021963001006643. doi: 10.1017/S0021963001006643.

22. Dubois B, Defontaines B, Deweer B, Malapani C, Pillon B. Cognitive and behavioral changes in patients with focal lesions of the basal ganglia. Behavioral neurology of movement disorders., New York, NY, US: Raven Press; (1995), p. 29–41.

23. Marin RS, Biedrzycki RC, Firinciogullari S. Reliability and validity of the apathy evaluation scale. Psychiatry Res. (1991) 38:143–62. doi: 10.1016/0165-1781(91)90040-V.

24. Hindmarch I, Lehfeld H, de Jongh P, Erzigkeit H. The Bayer Activities of Daily Living Scale (B-ADL). Dement Geriatr Cogn Disord. (1998) 9:20–6. doi: 10.1159/000051195.

25. Grace J, Malloy PH. Frontal systems behavior scale (FrSBe): Professional manual. Psychological Assessment Resources (PAR). (2001).

26. Yesavage JA, Brink TL, Rose TL, Lum O, Huang V, Adey M, et al. Development and validation of a geriatric depression screening scale: A preliminary report. J Psychiatr Res. (1982) 17:37–49. doi: 10.1016/0022-3956(82)90033-4.

27. Schroeter ML, Pawelke S, Bisenius S, Kynast J, Schuemberg K, Polyakova M, et al. A Modified Reading the Mind in the Eyes Test Predicts Behavioral Variant Frontotemporal Dementia Better Than Executive Function Tests. Front Aging Neurosci. (2018) 10:1–11. doi: 10.3389/fnagi.2018.00011.

28. Austin PC, White IR, Lee DS, van Buuren S. Missing Data in Clinical Research: A Tutorial on Multiple Imputation. Canadian Journal of Cardiology. (2021) 37:1322–31. doi: 10.1016/j.cjca.2020.11.010.

29. Hayati Rezvan P, Lee KJ, Simpson JA. The rise of multiple imputation: a review of the reporting and implementation of the method in medical research. BMC Med Res Methodol. (2015) 15:30. doi: 10.1186/s12874-015-0022-1.

30. Madley-Dowd P, Hughes R, Tilling K, Heron J. The proportion of missing data should not be used to guide decisions on multiple imputation. J Clin Epidemiol. (2019) 110:63–73. doi: 10.1016/j.jclinepi.2019.02.016.

31. Buuren S van, Groothuis-Oudshoorn K. mice : Multivariate Imputation by Chained Equations in R. J Stat Softw. (2011) 45:1–67. doi: 10.18637/jss.v045.i03.

32. Rubin DB. Inference and missing data. Biometrika. (1976) 63:581–92. doi: 10.1093/biomet/63.3.581.

33. van Buuren S. Flexible Imputation of Missing Data, Second Edition. Second Edition. | Boca Raton, Florida : CRC Press, [2019] |: Chapman and Hall/CRC; (2018). doi: 10.1201/9780429492259.

34. Şenbabaoğlu Y, Michailidis G, Li JZ. Critical limitations of consensus clustering in class discovery. Sci Rep. (2015) 4:6207. doi: 10.1038/srep06207.

35. Chiu D, Talhouk A. diceR: Diverse Cluster Ensemble in R (2021).

Table S1

Clinical characteristics across groups (without questionnaires). Mean scores (SD) across the variables included in the analysis is, separated by clinical diagnostic group.

Variables (range)	***Healthy Controls (N = 49)***	***Amnestic AD (N = 74)***	***bvFTD (N = 171)***	***svPPA (N = 52)***	***nfvPPA (N = 76)***	***lvPPA (N = 47)***
Age in Years	64.5 (9.3)	66.1 (9.7)	62.3 (9.3)	62.9 (7.9)	68.9 (8.3)	68.7 (6.1)
Years of Education in Years	15.0 (3.1)	13.5 (3.3)	13.5 (3.0)	14.7 (3.2)	13.1 (3.3)	13.3 (3.5)
CDR Sum of Boxes (0-12)	0.1 (0.2)	4.8 (2.9)	5.0 (3.1)	2.8 (1.7)	2.2 (1.8)	2.8 (2.3)
FTLD-CDR Sum of Boxes (0-18)	0.1 (0.3)	5.8 (3.4)	6.8 (3.9)	4.9 (2.4)	4.2 (2.4)	4.7 (2.8)
CDR: Memory (0-3)	0.1 (0.2)	1.4 (0.7)	0.8 (0.5)	0.8 (0.4)	0.5 (0.4)	0.9 (0.6)
CDR: Orientation (0-3)	0.0 (0.1)	0.7 (0.8)	0.4 (0.5)	0.1 (0.3)	0.2 (0.4)	0.4 (0.5)
CDR: Judgment, Problem Solving (0-3)	0.0 (0.0)	1.0 (0.7)	1.1 (0.7)	0.6 (0.6)	0.5 (0.5)	0.6 (0.6)
CDR: Community Affairs (0-3)	0.0 (0.1)	0.8 (0.6)	1.0 (0.7)	0.6 (0.4)	0.5 (0.4)	0.6 (0.6)
CDR: Home & Hobbies (0-3)	0.0 (0.0)	0.8 (0.6)	1.0 (0.7)	0.5 (0.4)	0.4 (0.4)	0.5 (0.5)
CDR: Personal Care (0-3)	0.0 (0.0)	0.2 (0.4)	0.6 (0.7)	0.1 (0.3)	0.1 (0.2)	0.1 (0.2)
FTLD-CDR: Behavior (0-3)	0.0 (0.1)	0.4 (0.4)	1.3 (0.7)	0.6 (0.6)	0.3 (0.4)	0.2 (0.4)
FTLD-CDR: Language (0-3)	0.0 (0.0)	0.6 (0.7)	0.5 (0.6)	1.4 (0.6)	1.6 (0.8)	1.7 (0.7)
CERAD: Semantic Fluency	26.5 (5.6)	12.2 (5.7)	12.8 (6.2)	9.0 (4.5)	11.0 (6.5)	10.7 (5.3)
CERAD: BNT (0-15)	14.9 (0.2)	12.4 (2.7)	13.2 (2.3)	6.5 (3.2)	12.1 (3.2)	10.9 (3.6)

Variables (range)	*Healthy Controls (N = 49)*	*Amnestic AD (N = 74)*	*bvFTD (N = 171)*	*svPPA (N = 52)*	*nfvPPA (N = 76)*	*lvPPA (N = 47)*
CERAD: MMSE (0-30)	29.2 (0.9)	22.9 (4.8)	25.4 (3.5)	23.8 (4.5)	24.7 (5.1)	22.3 (6.0)
CERAD: Total Wordlist (0-30)	22.6 (3.1)	11.4 (4.5)	15.2 (4.6)	14.5 (6.2)	15.1 (5.9)	12.0 (6.5)
CERAD: Wordlist 1 (0-10)	5.8 (1.4)	2.7 (1.6)	3.8 (1.7)	3.4 (1.8)	3.7 (1.9)	2.7 (2.1)
CERAD: Wordlist 2 (0-10)	7.8 (1.3)	4.1 (1.7)	5.3 (1.7)	5.2 (2.4)	5.2 (2.2)	4.2 (2.2)
CERAD: Wordlist 3 (0-10)	9.0 (1.0)	4.6 (1.9)	6.1 (1.9)	5.9 (2.5)	6.1 (2.2)	5.1 (2.7)
CERAD: Wordlist Recall (0-10)	8.1 (1.8)	2.4 (2.2)	4.4 (2.3)	3.8 (3.1)	5.1 (2.5)	4.1 (2.7)
CERAD: Wordlist Savings	89.8 (17.3)	46.8 (37.9)	69.3 (28.9)	53.3 (36.6)	82.2 (30.0)	76.8 (39.9)
CERAD: Wordlist Recognition (0-100)	99.2 (2.1)	84.4 (12.8)	88.7 (14.8)	83.4 (13.5)	91.7 (13.4)	91.4 (10.3)
CERAD: Wordlist Intrusions	0.3 (0.6)	2.8 (4.6)	1.6 (2.1)	1.7 (3.1)	0.9 (1.3)	1.1 (1.8)
CERAD: Figure Copy (0-11)	10.8 (0.7)	9.3 (2.1)	9.6 (1.8)	10.4 (1.3)	9.8 (1.8)	9.9 (1.3)
CERAD: Figure Recall (0-11)	9.7 (1.8)	3.5 (3.0)	6.0 (3.6)	6.8 (3.3)	7.6 (3.2)	6.0 (3.3)
CERAD: Figure Savings	89.9 (16.3)	36.2 (29.6)	61.1 (33.7)	65.5 (29.1)	75.8 (28.6)	59.1 (30.5)
CERAD: Phonemic Fluency	17.8 (4.3)	8.5 (4.4)	7.3 (4.5)	7.5 (3.8)	4.5 (3.7)	6.1 (3.4)
CERAD: TMT A (0-180)	37.6 (12.3)	86.9 (44.6)	73.6 (37.9)	54.6 (24.6)	85.8 (43.7)	78.0 (43.0)
CERAD: TMT B (0-300)	79.9 (32.3)	215.2 (75.9)	177.5 (81.8)	132.9 (67.1)	211.8 (78.4)	205.7 (78.0)
CERAD: TMT B/A	2.2 (0.6)	3.1 (1.1)	2.9 (1.3)	2.7 (1.4)	2.9 (0.9)	3.4 (1.1)
CERAD: TMT Errors	0.6 (1.0)	1.7 (2.6)	2.2 (2.3)	0.4 (0.8)	2.7 (2.7)	1.3 (1.4)

Variables (range)	*Healthy Controls (N = 49)*	*Amnestic AD (N = 74)*	*bvFTD (N = 171)*	*svPPA (N = 52)*	*nfvPPA (N = 76)*	*lvPPA (N = 47)*
WMS-R: Digit Span Forward (0-12)	8.7 (1.7)	5.8 (2.2)	6.2 (1.8)	6.5 (2.2)	4.5 (1.9)	3.8 (2.2)
WMS-R: Digit Span Backward (0-12)	7.1 (2.2)	4.1 (1.8)	4.1 (1.7)	5.2 (1.7)	3.5 (1.6)	3.3 (1.8)
WMS-R: Visual Span Forward (0-14)	8.0 (2.0)	5.3 (2.0)	5.9 (2.2)	7.8 (1.9)	6.2 (1.8)	5.6 (1.7)
WMS-R: Visual Span Backward (0-12)	7.3 (1.9)	4.2 (1.8)	5.1 (2.2)	6.9 (1.8)	5.1 (2.3)	4.9 (1.9)
Cookie Theft Task (0-20)	14.7 (2.8)	10.9 (3.5)	9.8 (3.5)	8.8 (3.7)	9.4 (3.9)	9.7 (3.4)
AAT: Token Test (0-50)	0.6 (1.1)	6.7 (7.0)	5.8 (6.9)	8.4 (10.4)	10.8 (9.5)	10.6 (9.0)
AAT: Written Language (0-90)	89.7 (0.7)	84.7 (6.3)	84.3 (10.5)	84.7 (6.0)	78.7 (15.5)	79.2 (13.0)
Repeat (0-10)	9.9 (0.3)	9.5 (1.2)	9.8 (0.7)	9.5 (0.9)	8.2 (2.5)	8.3 (2.2)
Point (0-10)	9.9 (0.4)	8.7 (1.5)	8.7 (1.6)	6.6 (2.4)	9.0 (1.2)	8.6 (1.7)
Stroop: Color Naming	72.8 (11.1)	43.7 (15.0)	45.9 (17.1)	50.4 (19.0)	31.6 (14.1)	34.8 (14.5)
Stroop: Word Reading	98.2 (10.2)	59.9 (18.4)	65.3 (21.4)	76.1 (21.8)	46.1 (18.3)	57.3 (18.7)
Stroop: Interference	41.2 (10.2)	17.1 (8.4)	20.5 (11.8)	27.6 (11.9)	16.1 (10.6)	14.3 (11.2)
Stroop: Error	0.4 (1.0)	2.6 (3.6)	3.0 (8.1)	2.7 (7.9)	1.6 (4.1)	2.5 (4.4)
H5PT: Total Correct	32.0 (8.0)	15.6 (6.3)	16.3 (10.0)	20.7 (8.4)	17.4 (8.3)	18.6 (8.2)
H5PT: Total	35.1 (7.9)	20.7 (9.6)	25.1 (13.8)	25.6 (10.2)	23.8 (10.5)	24.9 (13.3)

Variables (range)	*Healthy Controls (N = 49)*	*Amnestic AD (N = 74)*	*bvFTD (N = 171)*	*svPPA (N = 52)*	*nfvPPA (N = 76)*	*lvPPA (N = 47)*
H5PT: Percent Correct (0-100)	91.2 (12.6)	79.5 (18.4)	68.4 (27.2)	82.2 (20.6)	77.2 (22.4)	81.1 (19.8)
Cognitive Estimation (0-16)	12.7 (2.1)	8.8 (3.1)	9.4 (3.2)	9.2 (3.1)	9.4 (3.1)	9.5 (3.2)
Applause Sign (0-6)	0.1 (0.3)	0.1 (0.4)	0.3 (0.9)	0.3 (0.7)	0.5 (1.3)	0.1 (0.5)
RMET (0-24)	16.5 (2.7)	12.5 (3.6)	10.9 (3.7)	10.1 (2.6)	10.6 (2.7)	11.6 (3.8)

Note: Abbreviations: AAT Aachen aphasia test, AD Alzheimer's Disease, AES apathy evaluation scale, B.ADL Bayer activities of daily living, BNT Boston Naming Test, bvFTD behavioral variant frontotemporal dementia, CDR clinical dementia rating, CERAD Consortium to establish a registry for Alzheimer's Disease, FrSBe Frontal Systems Behavior Scale, FTLD-CDR frontotemporal lobar degeneration clinical dementia rating, GDS geriatric depression scale, H5PT Hamasch five point test, lvPPA logopenic variant primary progressive aphasia, MMSE mini-mental state examination, nfvPPA non-fluent variant primary progressive aphasia, RMET reading the mind in the eyes test, svPPA semantic variant primary progressive aphasia, TMT trail making test, WMS-R Wechsler memory scale revised.

Table S2

Group clinical characteristics across groups (with questionnaires). Mean scores (SD) across the variables included in the analysis, separated by clinical diagnostic group.

Variables (range)	*Healthy Controls (N = 49)*	*Amnestic AD (N = 70)*	*bvFTD (N = 173)*	*svPPA (N = 50)*	*nfvPPA (N = 72)*	*lvPPA (N = 48)*
Age in Years	64.5 (9.3)	66.4 (9.7)	62.3 (9.5)	63.2 (7.9)	68.7 (8.6)	68.4 (6.2)
Years of Education in Years	15.0 (3.1)	13.4 (3.4)	13.4 (2.9)	14.6 (3.3)	13.1 (3.4)	13.3 (3.4)
CDR Sum of Boxes (0-12)	0.1 (0.2)	4.7 (2.8)	5.1 (3.2)	2.8 (1.7)	2.2 (1.8)	2.8 (2.3)
FTLD-CDR Sum of Boxes (0-18)	0.1 (0.3)	5.7 (3.3)	6.9 (4.0)	4.8 (2.4)	4.2 (2.5)	4.7 (2.8)
CDR: Memory (0-3)	0.1 (0.2)	1.4 (0.7)	0.8 (0.6)	0.8 (0.4)	0.5 (0.5)	0.9 (0.6)
CDR: Orientation (0-3)	0.0 (0.1)	0.7 (0.8)	0.5 (0.5)	0.2 (0.3)	0.2 (0.4)	0.4 (0.5)
CDR: Judgment, Problem Solving (0-3)	0.0 (0.0)	1.0 (0.6)	1.2 (0.7)	0.6 (0.6)	0.5 (0.5)	0.6 (0.6)
CDR: Community Affairs (0-3)	0.0 (0.1)	0.8 (0.6)	1.0 (0.7)	0.6 (0.4)	0.5 (0.4)	0.6 (0.6)
CDR: Home & Hobbies (0-3)	0.0 (0.0)	0.8 (0.6)	1.0 (0.7)	0.5 (0.4)	0.4 (0.4)	0.5 (0.5)
CDR: Personal Care (0-3)	0.0 (0.0)	0.2 (0.4)	0.6 (0.7)	0.1 (0.3)	0.1 (0.3)	0.1 (0.2)
FTLD-CDR: Behavior (0-3)	0.0 (0.1)	0.3 (0.4)	1.3 (0.7)	0.6 (0.6)	0.3 (0.4)	0.2 (0.4)
FTLD-CDR: Language (0-3)	0.0 (0.0)	0.6 (0.7)	0.5 (0.6)	1.4 (0.6)	1.6 (0.7)	1.7 (0.7)
CERAD: Semantic Fluency	26.5 (5.6)	12.2 (5.7)	12.8 (6.2)	8.9 (4.5)	11.4 (6.6)	10.8 (5.3)
CERAD: BNT (0-15)	14.9 (0.2)	12.3 (2.8)	13.1 (2.4)	6.4 (3.1)	12.1 (3.2)	10.9 (3.5)
CERAD: MMSE (0-30)	29.2 (0.9)	22.9 (5.1)	25.3 (3.7)	23.7 (4.6)	24.9 (5.2)	22.2 (6.0)

Variables (range)	Healthy Controls (N = 49)	Amnestic AD (N = 70)	bvFTD (N = 173)	svPPA (N = 50)	nfvPPA (N = 72)	lvPPA (N = 48)
CERAD: Total Wordlist (0-30)	22.6 (3.1)	11.4 (4.7)	15.2 (4.5)	14.3 (5.8)	15.4 (6.0)	12.0 (6.5)
CERAD: Wordlist 1 (0-10)	5.8 (1.4)	2.7 (1.6)	3.8 (1.6)	3.3 (1.6)	3.8 (1.9)	2.7 (2.0)
CERAD: Wordlist 2 (0-10)	7.8 (1.3)	4.1 (1.8)	5.3 (1.6)	5.2 (2.3)	5.3 (2.3)	4.2 (2.2)
CERAD: Wordlist 3 (0-10)	9.0 (1.0)	4.7 (1.9)	6.1 (1.9)	5.9 (2.4)	6.2 (2.3)	5.1 (2.7)
CERAD: Wordlist Recall (0-10)	8.1 (1.8)	2.4 (2.2)	4.4 (2.2)	3.6 (3.0)	5.1 (2.6)	4.1 (2.7)
CERAD: Wordlist Savings	89.8 (17.3)	46.0 (38.2)	70.0 (28.8)	52.5 (36.4)	82.3 (30.8)	76.5 (39.5)
CERAD: Wordlist Recognition (0-100)	99.2 (2.1)	84.0 (13.0)	88.7 (14.7)	82.3 (14.0)	92.4 (12.1)	91.5 (10.2)
CERAD: Wordlist Intrusions	0.3 (0.6)	2.8 (4.7)	1.6 (2.1)	1.8 (3.1)	0.8 (1.3)	1.1 (1.7)
CERAD: Figure Copy (0-11)	10.8 (0.7)	9.4 (1.9)	9.5 (1.8)	10.3 (1.3)	9.8 (1.8)	9.8 (1.4)
CERAD: Figure Recall (0-11)	9.7 (1.8)	3.7 (3.0)	6.0 (3.6)	6.8 (3.3)	7.7 (3.1)	5.9 (3.2)
CERAD: Figure Savings	89.9 (16.3)	37.3 (29.6)	60.6 (34.1)	64.9 (29.5)	77.0 (28.0)	59.4 (30.2)
CERAD: Phonemic Fluency	17.8 (4.3)	8.6 (4.4)	7.3 (4.5)	7.3 (3.7)	4.8 (3.8)	6.1 (3.4)
CERAD: TMT A (0-180)	37.6 (12.3)	88.8 (46.0)	74.6 (39.5)	52.6 (20.9)	81.3 (40.7)	77.3 (42.8)
CERAD: TMT B (0-300)	79.9 (32.3)	213.5 (75.7)	177.9 (82.0)	136.8 (72.0)	211.7 (78.4)	208.5 (78.5)
CERAD: TMT B/A	2.2 (0.6)	3.1 (1.1)	3.0 (1.3)	2.8 (1.4)	3.0 (1.0)	3.5 (1.2)
CERAD: TMT Errors	0.6 (1.0)	1.7 (2.6)	2.2 (2.3)	0.4 (0.8)	2.6 (2.5)	1.3 (1.4)
WMS-R: Digit Span Forward (0-12)	8.7 (1.7)	5.7 (2.3)	6.1 (1.8)	6.5 (2.2)	4.5 (1.9)	3.8 (2.2)

Variables (range)	Healthy Controls (N = 49)	Amnestic AD (N = 70)	bvFTD (N = 173)	svPPA (N = 50)	nfvPPA (N = 72)	lvPPA (N = 48)
WMS-R: Digit Span Backward (0-12)	7.1 (2.2)	4.1 (1.8)	4.0 (1.7)	5.1 (1.7)	3.6 (1.6)	3.2 (1.8)
WMS-R: Visual Span Forward (0-14)	8.0 (2.0)	5.4 (2.0)	5.9 (2.2)	7.8 (1.9)	6.4 (1.6)	5.5 (1.7)
WMS-R: Visual Span Backward (0-12)	7.3 (1.9)	4.2 (1.8)	5.1 (2.3)	6.9 (1.8)	5.1 (2.4)	4.8 (1.9)
Cookie Theft Task (0-20)	14.7 (2.8)	10.9 (3.5)	9.9 (3.5)	8.7 (3.8)	9.6 (3.8)	9.7 (3.3)
AAT: Token Test (0-50)	0.6 (1.1)	6.9 (7.7)	6.0 (7.3)	8.9 (11.2)	10.4 (9.0)	11.0 (9.3)
AAT: Written Language (0-90)	89.7 (0.7)	84.3 (8.5)	83.9 (10.8)	84.5 (6.1)	79.9 (14.5)	79.4 (12.9)
Repeat (0-10)	9.9 (0.3)	9.4 (1.3)	9.7 (0.9)	9.5 (1.0)	8.4 (2.1)	8.3 (2.2)
Point (0-10)	9.9 (0.4)	8.7 (1.6)	8.7 (1.6)	6.5 (2.5)	9.1 (1.2)	8.6 (1.7)
Stroop: Color Naming	72.8 (11.1)	44.0 (14.9)	45.8 (17.0)	49.8 (18.5)	31.5 (14.2)	34.5 (14.5)
Stroop: Word Reading	98.2 (10.2)	59.8 (19.0)	65.4 (21.4)	75.2 (22.2)	45.8 (18.2)	57.6 (18.6)
Stroop: Interference	41.2 (10.2)	17.2 (8.5)	20.6 (11.7)	27.4 (12.1)	16.0 (10.5)	14.0 (11.2)
Stroop: Error	0.4 (1.0)	2.7 (3.7)	3.0 (8.0)	2.9 (8.0)	1.6 (4.1)	2.4 (4.3)
H5PT: Total Correct	32.0 (8.0)	15.6 (6.3)	16.4 (10.2)	20.5 (8.4)	17.9 (8.3)	18.5 (8.1)
H5PT: Total	35.1 (7.9)	20.3 (9.5)	25.2 (13.9)	25.4 (10.3)	23.3 (9.5)	24.8 (13.2)
H5PT: Percent Correct (0-100)	91.2 (12.6)	80.6 (17.6)	68.0 (26.8)	82.5 (20.9)	79.2 (20.4)	80.8 (19.6)
Cognitive Estimation (0-16)	12.7 (2.1)	8.9 (3.1)	9.4 (3.2)	9.1 (3.0)	9.4 (3.1)	9.5 (3.2)

Variables (range)	Healthy Controls (N = 49)	Amnestic AD (N = 70)	bvFTD (N = 173)	svPPA (N = 50)	nfvPPA (N = 72)	lvPPA (N = 48)
Applause Sign (0-6)	0.1 (0.3)	0.1 (0.4)	0.3 (1.0)	0.3 (0.7)	0.4 (1.1)	0.1 (0.5)
RMET (0-24)	16.5 (2.7)	12.4 (3.7)	10.9 (3.8)	10.0 (2.7)	10.6 (2.8)	11.7 (3.7)
AES (0-54)	7.4 (4.8)	20.1 (12.1)	34.6 (9.7)	17.7 (9.0)	16.7 (10.3)	15.9 (10.3)
B-ADL (1-10)	1.5 (0.5)	4.2 (2.3)	5.1 (2.4)	3.7 (2.1)	3.3 (1.9)	3.6 (2.1)
FrSBe: Executive Dysfunction (8-40)	13.8 (4.1)	21.5 (8.1)	26.6 (6.7)	18.5 (7.2)	15.0 (6.1)	18.4 (6.8)
FrSBe: Disinhibition (8-40)	14.3 (3.5)	12.8 (5.0)	17.5 (6.3)	14.2 (5.4)	10.6 (3.5)	12.7 (4.0)
FrSBe: Apathy (8-40)	11.7 (3.7)	18.0 (8.5)	27.8 (7.2)	17.5 (6.3)	16.7 (6.6)	16.3 (6.9)
GDS (0-15)	1.6 (1.8)	3.6 (2.5)	3.5 (3.1)	4.9 (3.6)	3.5 (2.6)	4.5 (3.0)

Note: Abbreviations: AAT Aachen aphasia test, AD Alzheimer's Disease, AES apathy evaluation scale, B.ADL Bayer activities of daily living, BNT Boston Naming Test, bvFTD behavioral variant frontotemporal dementia, CDR clinical dementia rating, CERAD Consortium to establish a registry for Alzheimer's Disease, FrSBe Frontal Systems Behavior Scale, FTLD-CDR frontotemporal lobar degeneration clinical dementia rating, GDS geriatric depression scale, H5PT Hamasch five point test, lvPPA logopenic variant primary progressive aphasia, MMSE mini-mental state examination, nfvPPA non-fluent variant primary progressive aphasia, RMET reading the mind in the eyes test, svPPA semantic variant primary progressive aphasia, TMT trail making test, WMS-R Wechsler memory scale revised

Table S3

Number (percentage) of missing values for each variable included in the analysis

	Excluding Questionnaires			*Including Questionnaires*		
Variable	***Patients***	***Healthy Controls***	***Total***	***Patients***	***Healthy Controls***	***Total***
Age in Years	0 (0%)	0 (0%)	0 (0%)	0 (0%)	0 (0%)	0 (0%)
Years of Education in Years	14 (3.33%)	2 (4.08%)	16 (3.41%)	17 (4.12%)	2 (4.08%)	19 (4.11%)
CDR Sum of Boxes	2 (0.48%)	0 (0%)	2 (0.43%)	10 (2.42%)	0 (0%)	10 (2.16%)
FTLD-CDR Sum of Boxes	5 (1.19%)	0 (0%)	5 (1.07%)	13 (3.15%)	0 (0%)	13 (2.81%)
CDR: Memory	0 (0%)	0 (0%)	0 (0%)	9 (2.18%)	0 (0%)	9 (1.95%)
CDR: Orientation	0 (0%)	0 (0%)	0 (0%)	9 (2.18%)	0 (0%)	9 (1.95%)
CDR: Judgment, Problem Solving	2 (0.48%)	0 (0%)	2 (0.43%)	10 (2.42%)	0 (0%)	10 (2.16%)
CDR: Community Affairs	1 (0.24%)	0 (0%)	1 (0.21%)	9 (2.18%)	0 (0%)	9 (1.95%)
CDR: Home & Hobbies	1 (0.24%)	0 (0%)	1 (0.21%)	9 (2.18%)	0 (0%)	9 (1.95%)
CDR: Personal Care	1 (0.24%)	0 (0%)	1 (0.21%)	9 (2.18%)	0 (0%)	9 (1.95%)
FTLD-CDR: Behavior	2 (0.48%)	0 (0%)	2 (0.43%)	11 (2.66%)	0 (0%)	11 (2.38%)
FTLD-CDR: Language	3 (0.71%)	0 (0%)	3 (0.64%)	12 (2.91%)	0 (0%)	12 (2.6%)
CERAD: Semantic Fluency	5 (1.19%)	0 (0%)	5 (1.07%)	5 (1.21%)	0 (0%)	5 (1.08%)
CERAD: BNT	1 (0.24%)	0 (0%)	1 (0.21%)	1 (0.24%)	0 (0%)	1 (0.22%)
CERAD: MMSE	1 (0.24%)	0 (0%)	1 (0.21%)	1 (0.24%)	0 (0%)	1 (0.22%)

	Excluding Questionnaires			*Including Questionnaires*		
Variable	***Patients***	***Healthy Controls***	***Total***	***Patients***	***Healthy Controls***	***Total***
CERAD: Total Wordlist	0 (0%)	0 (0%)	0 (0%)	0 (0%)	0 (0%)	0 (0%)
CERAD: Wordlist 1	0 (0%)	0 (0%)	0 (0%)	0 (0%)	0 (0%)	0 (0%)
CERAD: Wordlist 2	0 (0%)	0 (0%)	0 (0%)	0 (0%)	0 (0%)	0 (0%)
CERAD: Wordlist 3	0 (0%)	0 (0%)	0 (0%)	0 (0%)	0 (0%)	0 (0%)
CERAD: Wordlist Recall	2 (0.48%)	0 (0%)	2 (0.43%)	1 (0.24%)	0 (0%)	1 (0.22%)
CERAD: Wordlist Savings	8 (1.9%)	0 (0%)	8 (1.71%)	7 (1.69%)	0 (0%)	7 (1.52%)
CERAD: Wordlist Recognition	3 (0.71%)	0 (0%)	3 (0.64%)	2 (0.48%)	0 (0%)	2 (0.43%)
CERAD: Wordlist Intrusions	2 (0.48%)	0 (0%)	2 (0.43%)	1 (0.24%)	0 (0%)	1 (0.22%)
CERAD: Figure Copy	1 (0.24%)	0 (0%)	1 (0.21%)	0 (0%)	0 (0%)	0 (0%)
CERAD: Figure Recall	3 (0.71%)	0 (0%)	3 (0.64%)	3 (0.73%)	0 (0%)	3 (0.65%)
CERAD: Figure Savings	3 (0.71%)	0 (0%)	3 (0.64%)	3 (0.73%)	0 (0%)	3 (0.65%)
CERAD: Phonemic Fluency	15 (3.57%)	0 (0%)	15 (3.2%)	13 (3.15%)	0 (0%)	13 (2.81%)
CERAD: TMT A	15 (3.57%)	0 (0%)	15 (3.2%)	11 (2.66%)	0 (0%)	11 (2.38%)
CERAD: TMT B	106 (25.24%)	0 (0%)	106 (22.6%)	98 (23.73%)	0 (0%)	98 (21.21%)
CERAD: TMT B/A	104 (24.76%)	0 (0%)	104 (22.17%)	96 (23.24%)	0 (0%)	96 (20.78%)
CERAD: TMT Errors	119 (28.33%)	0 (0%)	119 (25.37%)	109 (26.39%)	0 (0%)	109 (23.59%)
WMS-R: Digit Span Forward	3 (0.71%)	0 (0%)	3 (0.64%)	3 (0.73%)	0 (0%)	3 (0.65%)
WMS-R: Digit Span Backward	6 (1.43%)	0 (0%)	6 (1.28%)	6 (1.45%)	0 (0%)	6 (1.3%)

	Excluding Questionnaires			*Including Questionnaires*		
Variable	***Patients***	***Healthy Controls***	***Total***	***Patients***	***Healthy Controls***	***Total***
WMS-R: Visual Span Forward	17 (4.05%)	0 (0%)	17 (3.62%)	17 (4.12%)	0 (0%)	17 (3.68%)
WMS-R: Visual Span Backward	24 (5.71%)	0 (0%)	24 (5.12%)	24 (5.81%)	0 (0%)	24 (5.19%)
Cookie Theft Task	62 (14.76%)	14 (28.57%)	76 (16.2%)	57 (13.8%)	14 (28.57%)	71 (15.37%)
AAT: Token Test	30 (7.14%)	1 (2.04%)	31 (6.61%)	26 (6.3%)	1 (2.04%)	27 (5.84%)
AAT: Written Language	40 (9.52%)	1 (2.04%)	41 (8.74%)	33 (7.99%)	1 (2.04%)	34 (7.36%)
Repeat	20 (4.76%)	2 (4.08%)	22 (4.69%)	16 (3.87%)	2 (4.08%)	18 (3.9%)
Point	19 (4.52%)	2 (4.08%)	21 (4.48%)	16 (3.87%)	2 (4.08%)	18 (3.9%)
Stroop: Color Naming	36 (8.57%)	1 (2.04%)	37 (7.89%)	29 (7.02%)	1 (2.04%)	30 (6.49%)
Stroop: Word Reading	35 (8.33%)	3 (6.12%)	38 (8.1%)	28 (6.78%)	3 (6.12%)	31 (6.71%)
Stroop: Interference	70 (16.67%)	1 (2.04%)	71 (15.14%)	64 (15.5%)	1 (2.04%)	65 (14.07%)
Stroop: Error	70 (16.67%)	1 (2.04%)	71 (15.14%)	64 (15.5%)	1 (2.04%)	65 (14.07%)
H5PT: Total Correct	14 (3.33%)	0 (0%)	14 (2.99%)	14 (3.39%)	0 (0%)	14 (3.03%)
H5PT: Total	15 (3.57%)	0 (0%)	15 (3.2%)	15 (3.63%)	0 (0%)	15 (3.25%)
H5PT: Percent Correct	14 (3.33%)	0 (0%)	14 (2.99%)	14 (3.39%)	0 (0%)	14 (3.03%)
Cognitive Estimation	34 (8.1%)	0 (0%)	34 (7.25%)	32 (7.75%)	0 (0%)	32 (6.93%)
Applause Sign	34 (8.1%)	0 (0%)	34 (7.25%)	33 (7.99%)	0 (0%)	33 (7.14%)
RMET	66 (15.71%)	1 (2.04%)	67 (14.29%)	58 (14.04%)	1 (2.04%)	59 (12.77%)
AES	-	-	-	91 (22.03%)	13 (26.53%)	104 (22.51%)

	Excluding Questionnaires			Including Questionnaires		
Variable	***Patients***	***Healthy Controls***	***Total***	***Patients***	***Healthy Controls***	***Total***
B-ADL	-	-	-	88 (21.31%)	13 (26.53%)	101 (21.86%)
FrSBe: Executive Dysfunction	-	-	-	94 (22.76%)	10 (20.41%)	104 (22.51%)
FrSBe: Disinhibition	-	-	-	92 (22.28%)	10 (20.41%)	102 (22.08%)
FrSBe: Apathy	-	-	-	84 (20.34%)	8 (16.33%)	92 (19.91%)
GDS	-	-	-	103 (24.94%)	15 (30.61%)	118 (25.54%)

Note: Abbreviations: AAT Aachen aphasia test, AES apathy evaluation scale, B.ADL Bayer activities of daily living, BNT Boston Naming Test, CDR clinical dementia rating, CERAD Consortium to establish a registry for Alzheimer's Disease, FrSBe Frontal Systems Behavior Scale, FTLD-CDR frontotemporal lobar degeneration clinical dementia rating, GDS geriatric depression scale, H5PT Hamasch five point test, MMSE mini-mental state examination, RMET reading the mind in the eyes test, TMT trail making test, WMS-R Wechsler memory scale revised

Table S4

Cramer's V across all analyses performed, split by amount of clusters.

Amount of clusters	*Mean (SD)*
2	0.96 (0.03)
3	0.9 (0.11)
4	0.92 (0.05)
5	0.86 (0.09)
6	0.86 (0.1)
7	0.87 (0.09)
8	0.86 (0.09)
9	0.86 (0.08)

Table S5

Cramer's V across all analyses performed, split by groups compared.

Groups included	*Mean (SD)*
All Groups	0.9 (0.07)
All Patient Groups	0.86 (0.11)
PPA and bvFTD	0.88 (0.09)
PPA	0.89 (0.08)
Amnestic AD and bvFTD	0.88 (0.1)

Note: Abbreviations: AD Alzheimer's Disease, bvFTD behavioral variant frontotemporal dementia, PPA primary progressive aphasia.

Table S6

Proportion of ambiguous clustering (PAC) across all analyses performed, split by amount of clusters.

Amount of clusters	*Mean (SD)*
2	0.08 (0.04)
3	0.17 (0.1)
4	0.14 (0.05)
5	0.22 (0.08)
6	0.17 (0.07)
7	0.15 (0.05)
8	0.14 (0.04)
9	0.12 (0.03)

Table S7

Proportion of ambiguous clustering (PAC) across all analyses performed, split by groups compared.

Groups included	***Mean (SD)***
All Groups	0.12 (0.05)
All Patient Groups	0.17 (0.08)
PPA and bvFTD	0.17 (0.09)
PPA	0.13 (0.05)
Amnestic AD and bvFTD	0.15 (0.07)

Note: Abbreviations: AD Alzheimer's Disease, bvFTD behavioral variant frontotemporal dementia, PPA primary progressive aphasia.

Figure S1

Percentage of data present across all variables, split by participant group.

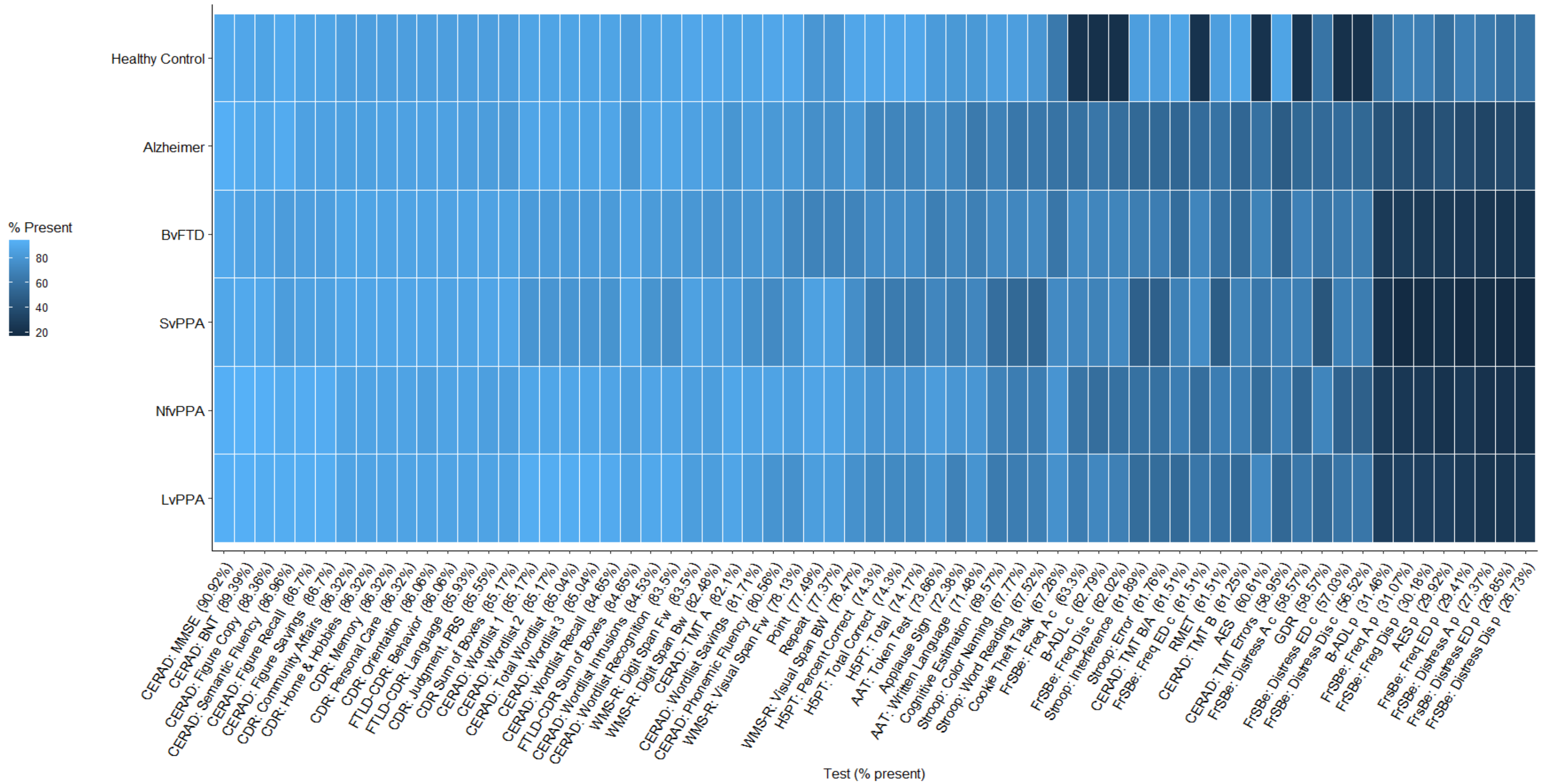

Note: Abbreviations: AAT Aachen aphasia test, AES apathy evaluation scale, B.ADL Bayer activities of daily living, BNT Boston Naming Test, BvFTD behavioral variant frontotemporal dementia, CDR clinical dementia rating, CERAD Consortium to establish a registry for Alzheimer's Disease, FrSBe Frontal Systems Behavior Scale, FTLD-CDR frontotemporal lobar degeneration clinical dementia rating, GDS geriatric depression scale, H5PT Hamasch five point test, LvPPA logopenic variant primary progressive aphasia, MMSE mini-mental state examination, NfvPPA non-

fluent variant primary progressive aphasia, RMET reading the mind in the eyes test, SvPPA semantic variant primary progressive aphasia, TMT trail making test, WMS-R Wechsler memory scale revised

Figure S2

Number of participants with specified percentage of missingness. ***(A)*** *count,* ***(B)*** *density.*

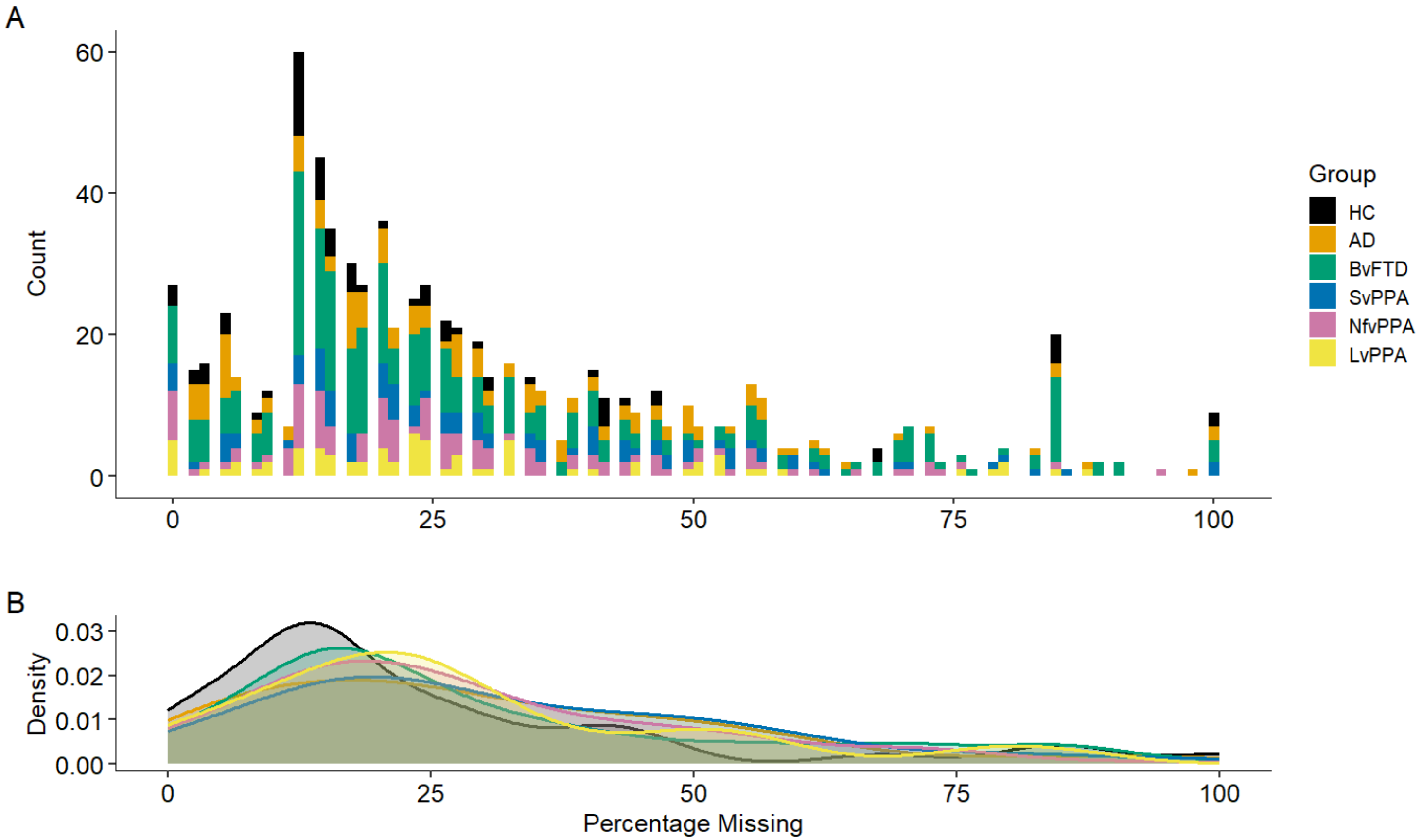

Note: Abbreviations: AD amnestic Alzheimer's Disease, BvFTD behavioral variant frontotemporal dementia, HC Healthy Controls, LvPPA logopenic variant primary progressive aphasia, NfvPPA non-fluent variant primary progressive aphasia, SvPPA semantic variant primary progressive aphasia

Figure S3

Density distribution of Cramer's V

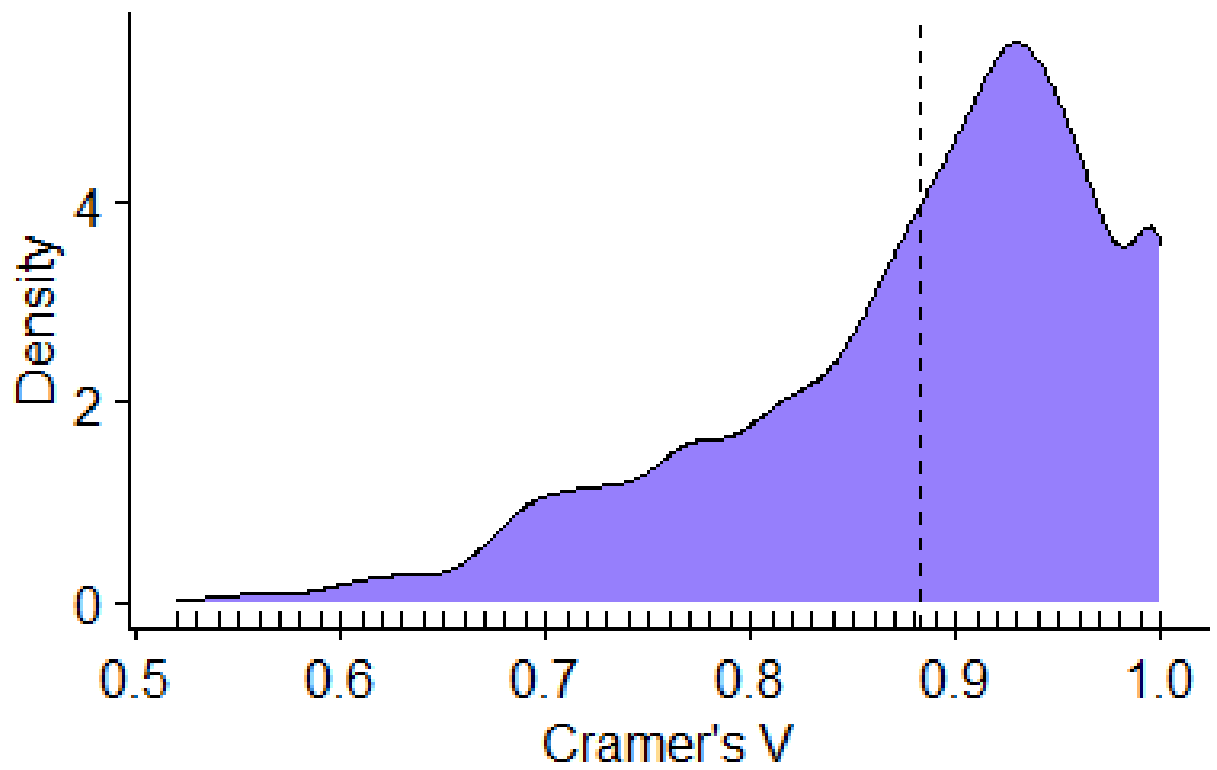

Figure S4

Density distribution of proportion of ambiguous clustering (PAC)

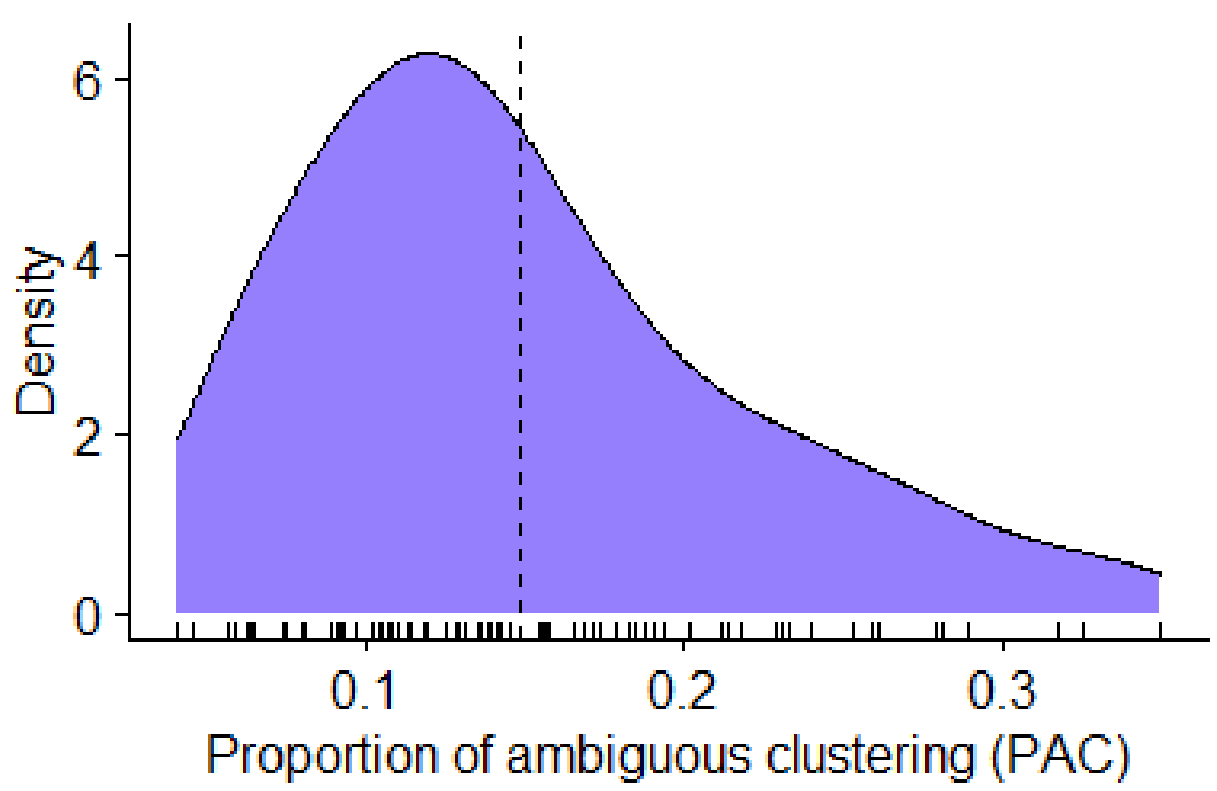

Figure S5

Composition of clusters by participant group in the analysis (excluding questionnaires). Analysis was repeated for cluster sizes 2-9 (left to right). Additionally, the analysis was repeated including specified participant groups **(A)** all groups, **(B)** all patient groups, **(C)** bvFTD and PPA groups, **(D)** PPA groups, **(E)** bvFTD and amnestic AD. Percentages displayed in the plot correspond to within-cluster proportions and were annotated for the largest group within a cluster. Mean and standard deviation of the proportion of ambiguous clustering (PAC) are shown for the group comparisons (A to E) and the number of clusters (k = 2-9) on the right and bottom of the plot, respectively.

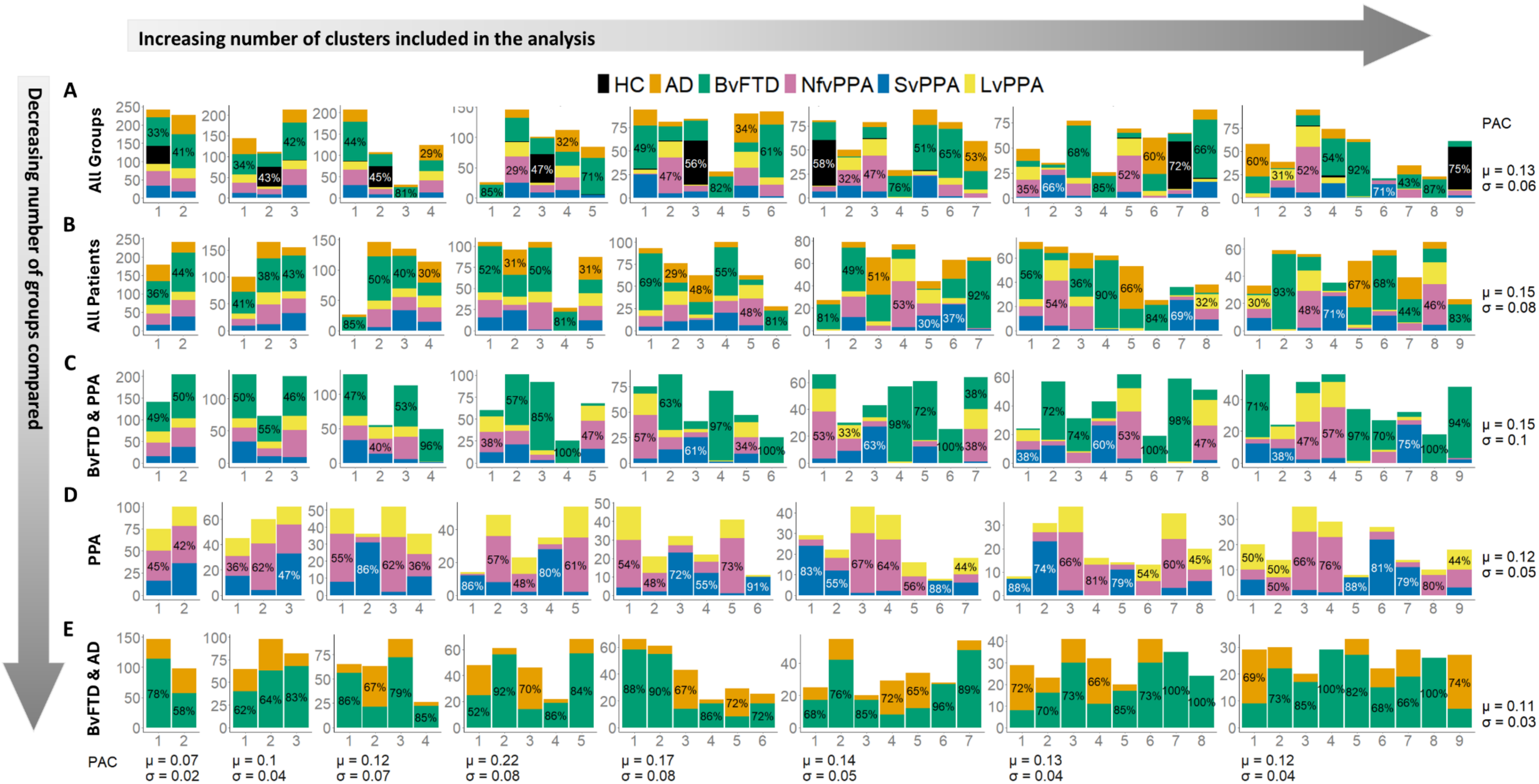
Increasing number of clusters included in the analysis
Decreasing number of groups compared
HC AD BvFTD NfvPPA SvPPA LvPPA
A All Groups
B All Patients
C BvFTD & PPA
D PPA
E BvFTD & AD
PAC
μ = 0.13
σ = 0.06
μ = 0.15
σ = 0.08
μ = 0.15
σ = 0.1
μ = 0.12
σ = 0.05
μ = 0.11
σ = 0.03
PAC
μ = 0.07
σ = 0.02
μ = 0.1
σ = 0.04
μ = 0.12
σ = 0.07
μ = 0.22
σ = 0.08
μ = 0.17
σ = 0.08
μ = 0.14
σ = 0.05
μ = 0.13
σ = 0.04
μ = 0.12
σ = 0.04

Note: Abbreviations: AD amnestic Alzheimer's Disease, BvFTD behavioral variant frontotemporal dementia, HC healthy controls, LvPPA logopenic variant primary progressive aphasia, NfvPPA non-fluent variant primary progressive aphasia, PAC proportion of ambiguous clustering, SvPPA semantic variant primary progressive aphasia.

Figure S6

Representation of the data used for the clustering analysis using the results from Principal Component Analysis (PCA). Participant group is indicated by the color of the points in the plots. The x-axes and the y-axes show the first and second principal components, respectively. The axes labels indicate the minimum and maximum percentage of explained variance of the respective principal components, varying due to the ten imputations performed. Plots **A** to **E** show the results without questionnaires and plots **F** to **J** the results from the analysis with the questionnaires. Additionally, separate plots show results for all participants (**A**, **F**), all patients (**B**, **G**), bvFTD and PPA (**C**, **H**), PPA (**D**, **I**) and bvFTD and amnestic AD (**E**, **J**)

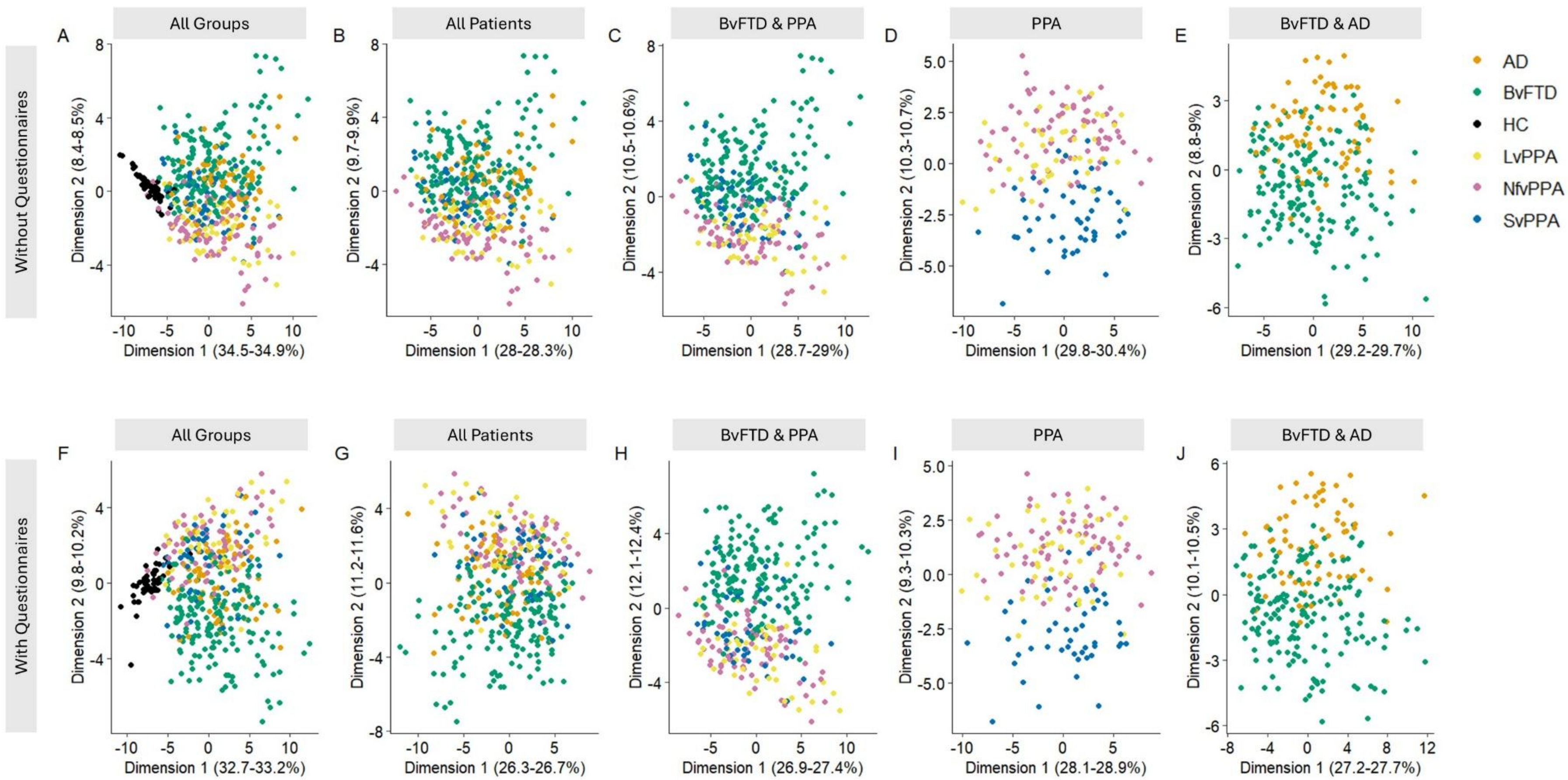

Note: Abbreviations: AD amnestic Alzheimer's Disease, BvFTD behavioral variant frontotemporal dementia, HC healthy controls, LvPPA logopenic variant primary progressive aphasia, NfvPPA non-fluent variant primary progressive aphasia, SvPPA semantic variant primary progressive aphasia.

In the following Figures S7 to S15 show radar plots representing cluster centers for each of the analyses performed. For simplification the radar plots selected correspond to the ones with best adjusted mutual information (AMI), that was used for external validation of clustering results with diagnostic groups contained in the data.

- Figure S7: all groups excluding questionnaires, number of clusters set to 9
- Figure S8: all groups including questionnaires, number of clusters set to 9
- Figure S9: all patients excluding questionnaires, number of clusters set to 7
- Figure S10: all patients including questionnaires, number of clusters set to 8
- Figure S11: BvFTD and PPA excluding questionnaires, number of clusters set to 6
- Figure S12: PPA excluding questionnaires, number of clusters set to 4
- Figure S13: PPA including questionnaires, number of clusters set to 5
- Figure S14: BvFTD and amnestic AD excluding questionnaires, number of clusters set to 4
- Figure S15: BvFTD and amnestic AD including questionnaires, number of clusters set to 3

Figure S7

Neuropsychological profiles of cluster results (excluding questionnaires, analysis including all groups, k = 9). Example plot representing neuropsychological profiles for the analysis including all groups, excluding questionnaires and limiting the analysis to k = 9 clusters. For the analysis scores were scaled to vary between -1 and 1. For visualization purposes only, cluster centers were z-standardized. Scores more at the inside of the radar plot correspond to worse performance. **(A)** Radar Plot indicating z-standardized cluster centers for clusters 1-9. **(B)** Box plot indicating groups within clusters 1-9. Percentages displayed in the plot correspond to within-cluster proportions and were annotated for the largest group within a cluster.

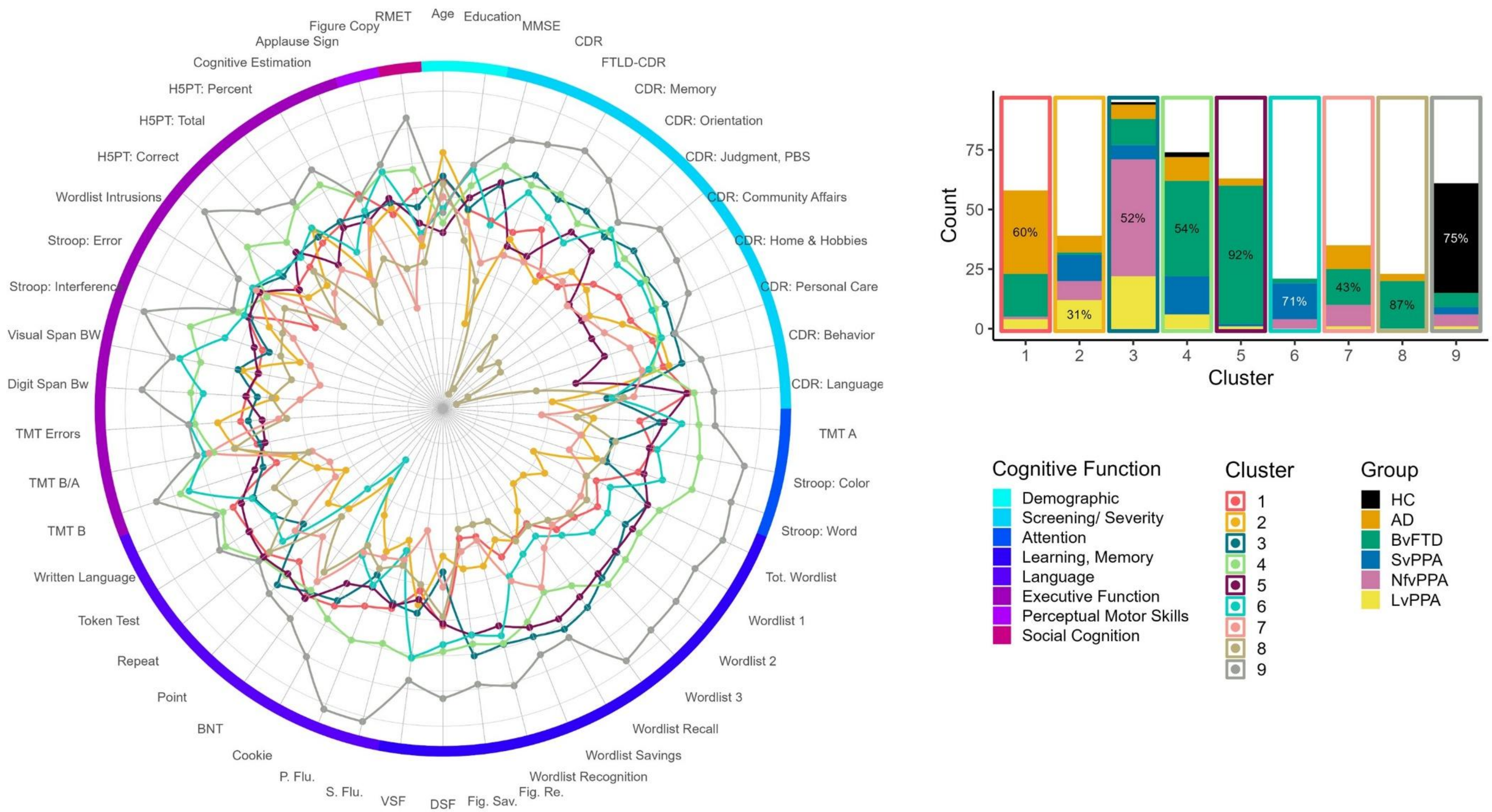

Abbreviations: AD amnestic Alzheimer's Disease, BNT Boston Naming Test, BvFTD behavioral variant frontotemporal dementia, CDR Clinical Dementia Rating Scale, DSF Digit span forward, Fig. Re. Figure Recall (from CERAD battery), Fig. Sav. Figure savings (from CERAD battery), FTLD-CDR Frontotemporal Lobar Degeneration Clinical Dementia Rating Scale, H5PT Hamasch five point Test, HC healthy controls, LvPPA logopenic variant primary progressive aphasia, MMSE Mini-Mental State Examination, NfvPPA non-fluent variant

primary progressive aphasia, TMT Trail Making Test, P. Flu. Phonematic fluency, RMET Reading the mind in the eyes test, S. Flu. Semantic fluency, SvPPA semantic variant primary progressive aphasia, VSF visual span forward.

Figure S8

Neuropsychological profiles of cluster results (including questionnaires, analysis including all groups, k = 9). Example plot representing neuropsychological profiles for the analysis including all groups, including questionnaires and limiting the analysis to k = 9 clusters. For the analysis scores were scaled to vary between -1 and 1. For visualization purposes only, cluster centers were z-standardized. Scores more at the inside of the radar plot correspond to worse performance. **(A)** Radar Plot indicating z-standardized cluster centers for clusters 1-9. **(B)** Box plot indicating groups within clusters 1-9. Percentages displayed in the plot correspond to within-cluster proportions and were annotated for the largest group within a cluster.

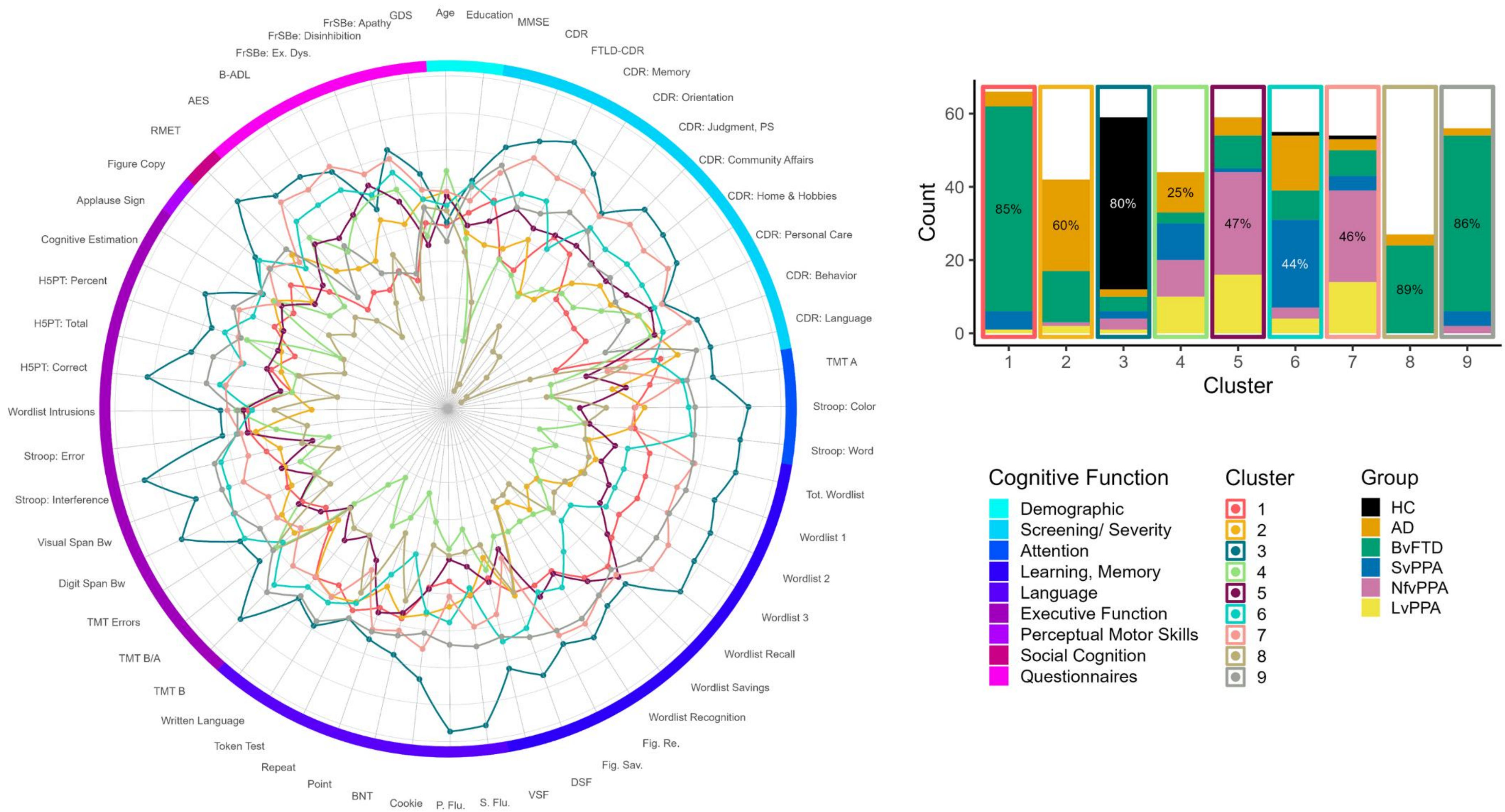

Abbreviations: AD amnestic Alzheimer's Disease, AES Apathy Evaluation Scale, B-ADL Bayer Activities of Daily Living, BNT Boston Naming Test, BvFTD behavioral variant frontotemporal dementia, CDR Clinical Dementia Rating Scale, DSF Digit span forward, Ex. Dys. Executive Dysfunction, Fig. Re. Figure Recall (from CERAD battery), Fig. Sav. Figure savings (from CERAD battery), FrSBe Frontal Systems Behavior Scale, FTLD-CDR Frontotemporal Lobar Degeneration Clinical Dementia Rating Scale, GDS Geriatric Depression Scale, H5PT Hamasch five point Test, HC healthy controls, LvPPA logopenic variant primary progressive aphasia, MMSE Mini-Mental State

Examination, NfvPPA non-fluent variant primary progressive aphasia, TMT Trail Making Test, P. Flu. Phonematic fluency, RMET Reading the mind in the eyes test, S. Flu. Semantic fluency, SvPPA semantic variant primary progressive aphasia, VSF visual span forward.

Figure S9

Neuropsychological profiles of cluster results (excluding questionnaires, analysis including all patient groups, k = 7). Example plot representing neuropsychological profiles for the analysis including all patient groups, excluding questionnaires and limiting the analysis to k = 7 clusters. For the analysis scores were scaled to vary between -1 and 1. For visualization purposes only, cluster centers were z-standardized. Scores more at the inside of the radar plot correspond to worse performance. **(A)** Radar Plot indicating z-standardized cluster centers for clusters 1-9. **(B)** Box plot indicating groups within clusters 1-7. Percentages displayed in the plot correspond to within-cluster proportions and were annotated for the largest group within a cluster.

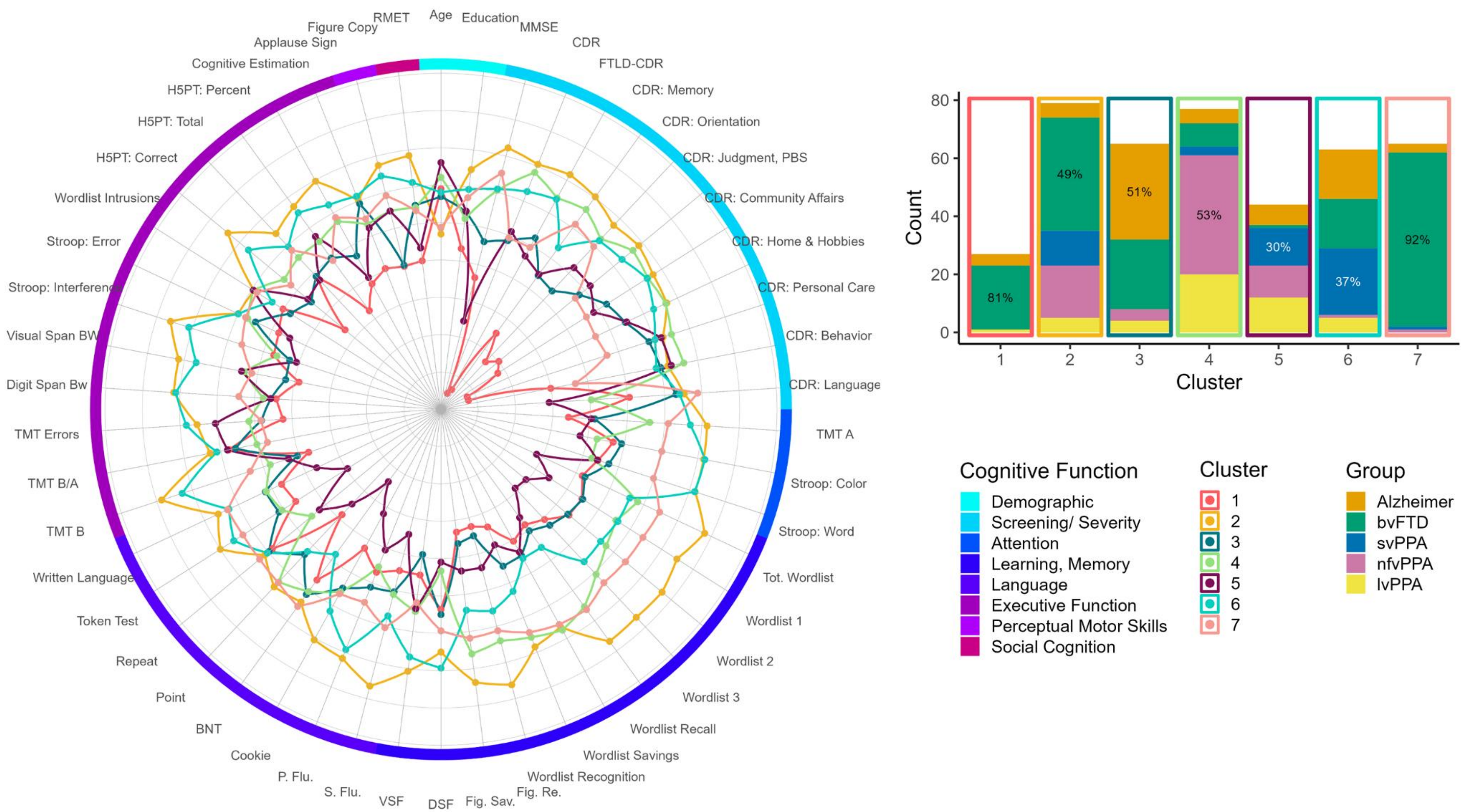

Abbreviations: AD amnestic Alzheimer’s Disease, BNT Boston Naming Test, BvFTD behavioral variant frontotemporal dementia, CDR Clinical Dementia Rating Scale, DSF Digit span forward, Fig. Re. Figure Recall (from CERAD battery), Fig. Sav. Figure savings (from CERAD battery), FTLD-CDR Frontotemporal Lobar Degeneration Clinical Dementia Rating Scale, H5PT Hamasch five point Test, LvPPA logopenic variant primary progressive aphasia, MMSE Mini-Mental State Examination, NfvPPA non-fluent variant primary progressive aphasia, TMT Trail Making Test, P. Flu. Phonematic fluency, RMET Reading the mind in the eyes test, S. Flu. Semantic fluency, SvPPA semantic variant primary progressive aphasia, VSF visual span forward.

Figure S10

Neuropsychological profiles of cluster results (including questionnaires, analysis including all patient groups, k = 8). Example plot representing neuropsychological profiles for the analysis including all patient groups, including questionnaires and limiting the analysis to k = 8 clusters. For the analysis scores were scaled to vary between -1 and 1. For visualization purposes only, cluster centers were z-standardized. Scores more at the inside of the radar plot correspond to worse performance. **(A)** Radar Plot indicating z-standardized cluster centers for clusters 1-9. **(B)** Box plot indicating groups within clusters 1-8. Percentages displayed in the plot correspond to within-cluster proportions and were annotated for the largest group within a cluster.

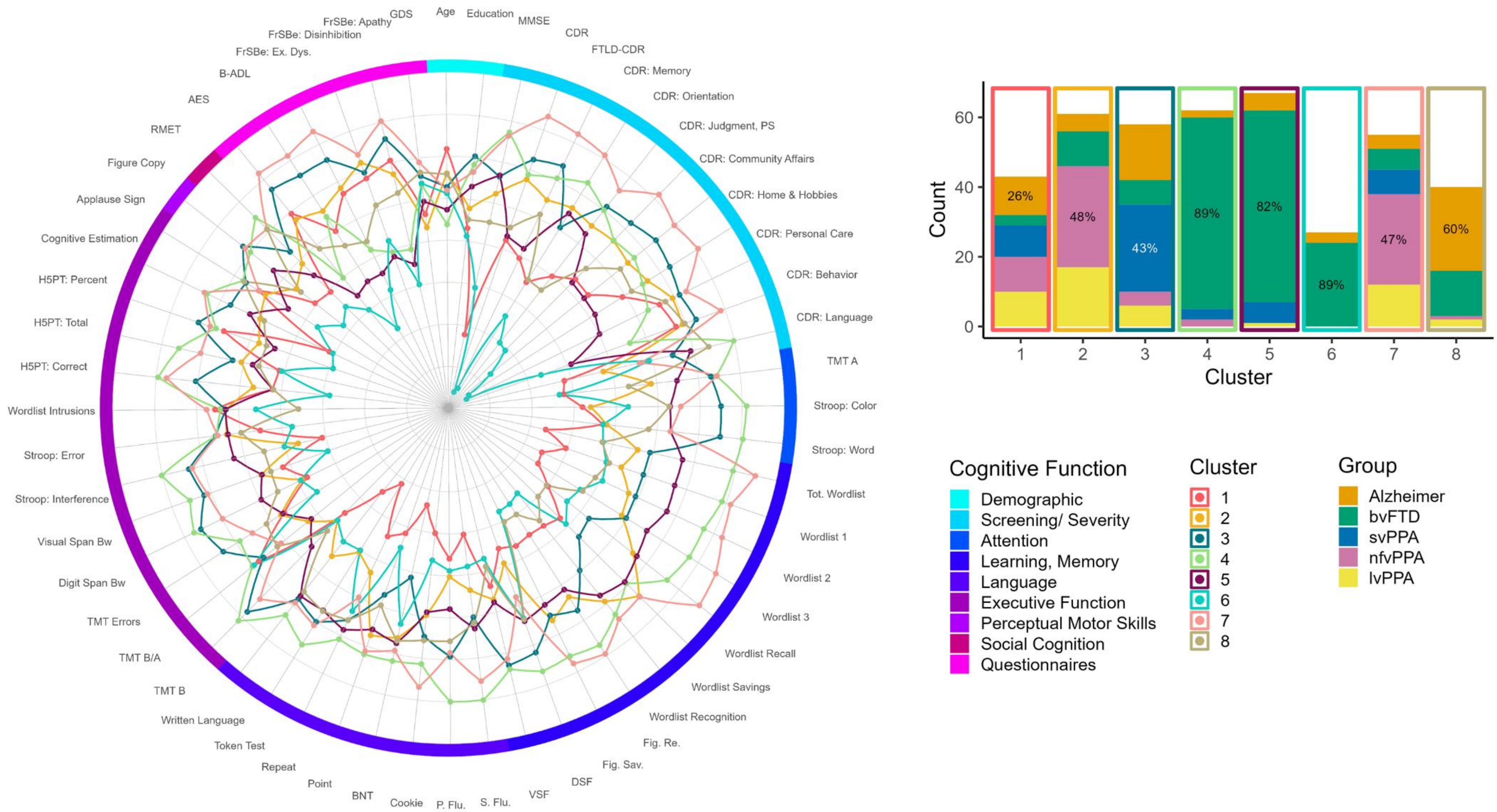

Abbreviations: AD amnestic Alzheimer's Disease, AES Apathy Evaluation Scale, B-ADL Bayer Activities of Daily Living, BNT Boston Naming Test, BvFTD behavioral variant frontotemporal dementia, CDR Clinical Dementia Rating Scale, DSF Digit span forward, Ex. Dys. Executive Dysfunction, Fig. Re. Figure Recall (from CERAD battery), Fig. Sav. Figure savings (from CERAD battery), FrSBe Frontal Systems Behavior Scale, FTLD-CDR Frontotemporal Lobar Degeneration Clinical Dementia Rating Scale, GDS Geriatric Depression Scale, H5PT Hamasch five point Test, LvPPA logopenic variant primary progressive aphasia, MMSE Mini-Mental State Examination, NfvPPA

non-fluent variant primary progressive aphasia, TMT Trail Making Test, P. Flu. Phonematic fluency, RMET Reading the mind in the eyes test, S. Flu. Semantic fluency, SvPPA semantic variant primary progressive aphasia, VSF visual span forward.

Figure S11

Neuropsychological profiles of cluster results (excluding questionnaires, analysis including bvFTD and the PPAs, k = 6). Example plot representing neuropsychological profiles for the analysis including patients diagnosed with bvFTD or one of the PPAs, excluding questionnaires and limiting the analysis to k = 6 clusters. For the analysis scores were scaled to vary between -1 and 1. For visualization purposes only, cluster centers were z-standardized. Scores more at the inside of the radar plot correspond to worse performance. **(A)** Radar Plot indicating z-standardized cluster centers for clusters 1-6. **(B)** Box plot indicating groups within clusters 1-9. Percentages displayed in the plot correspond to within-cluster proportions and were annotated for the largest group within a cluster.

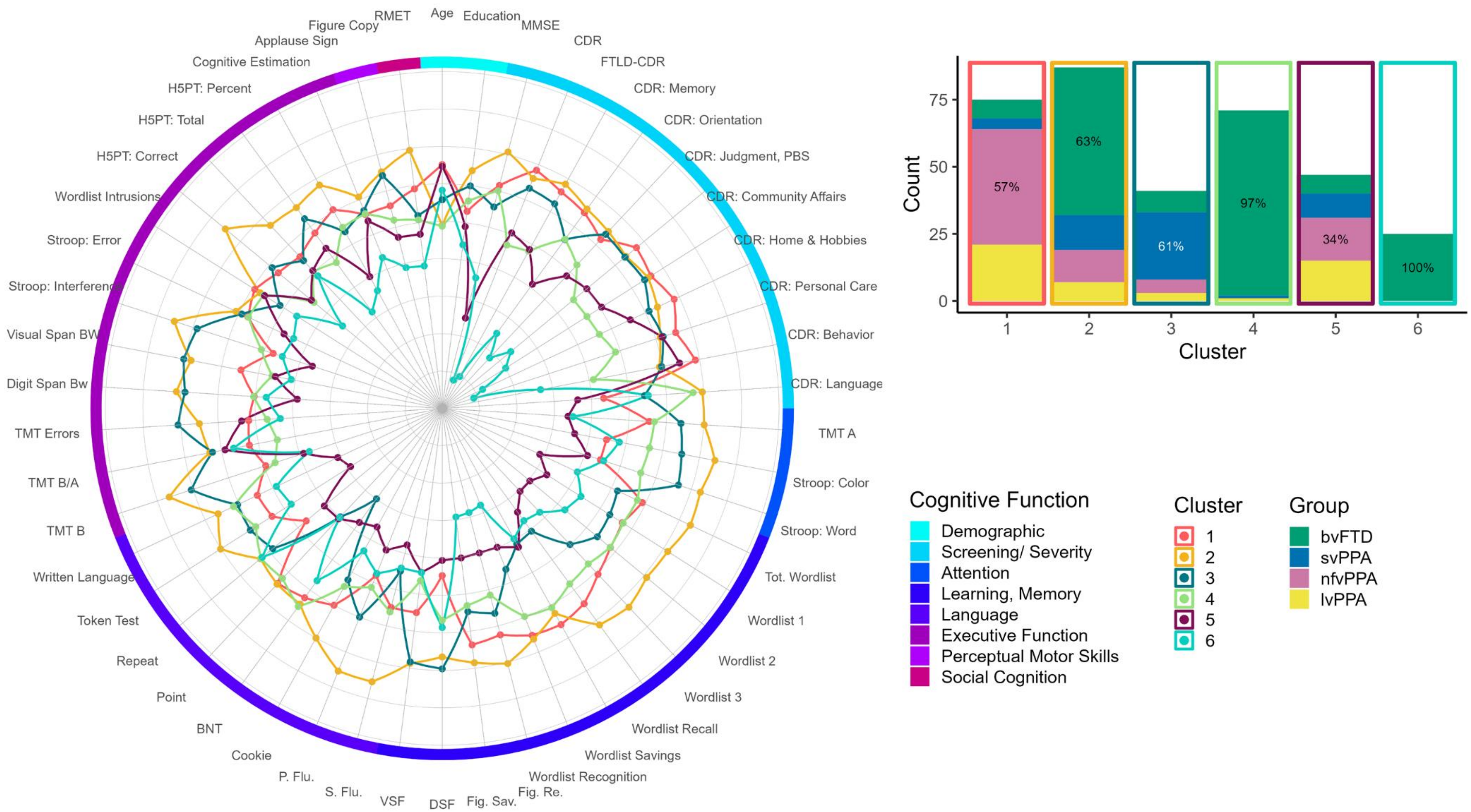

Abbreviations: BNT Boston Naming Test, BvFTD behavioral variant frontotemporal dementia, CDR Clinical Dementia Rating Scale, DSF Digit span forward, Fig. Re. Figure Recall (from CERAD battery), Fig. Sav. Figure savings (from CERAD battery), FTLD-CDR Frontotemporal Lobar Degeneration Clinical Dementia Rating Scale, H5PT Hamasch five point Test, LvPPA logopenic variant primary progressive aphasia, MMSE Mini-Mental State Examination, NfvPPA non-fluent variant primary progressive aphasia, TMT Trail Making Test, P. Flu. Phonematic fluency, RMET Reading the mind in the eyes test, S. Flu. Semantic fluency, SvPPA semantic variant primary progressive aphasia, VSF visual span forward.

Figure S12

Neuropsychological profiles of cluster results (excluding questionnaires, analysis including the PPAs, k = 4). Example plot representing neuropsychological profiles for the analysis including patients diagnosed with one of the PPA variants, excluding questionnaires and limiting the analysis to k = 4 clusters. For the analysis scores were scaled to vary between -1 and 1. For visualization purposes only, cluster centers were z-standardized. Scores more at the inside of the radar plot correspond to worse performance. **(A)** Radar Plot indicating z-standardized cluster centers for clusters 1-9. **(B)** Box plot indicating groups within clusters 1-4. Percentages displayed in the plot correspond to within-cluster proportions and were annotated for the largest group within a cluster.

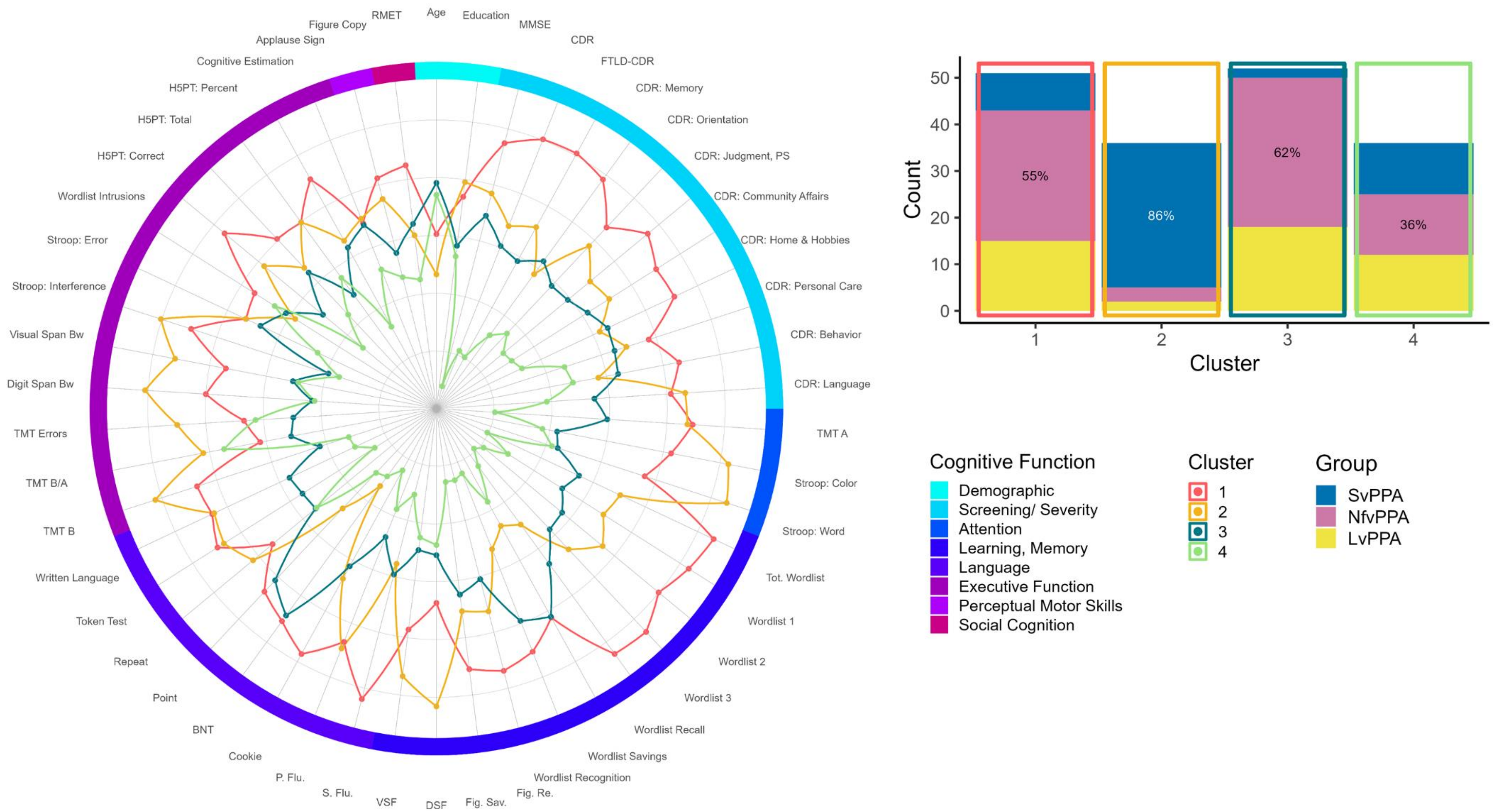

Abbreviations: BNT Boston Naming Test, CDR Clinical Dementia Rating Scale, DSF Digit span forward, Fig. Re. Figure Recall (from CERAD battery), Fig. Sav. Figure savings (from CERAD battery), FTLD-CDR Frontotemporal Lobar Degeneration Clinical Dementia Rating Scale, H5PT Hamasch five point Test, LvPPA logopenic variant primary progressive aphasia, MMSE Mini-Mental State Examination, NfvPPA non-fluent variant primary progressive aphasia, TMT Trail Making Test, P. Flu. Phonematic fluency, RMET Reading the mind in the eyes test, S. Flu. Semantic fluency, SvPPA semantic variant primary progressive aphasia, VSF visual span forward.

Figure S13

Neuropsychological profiles of cluster results (including questionnaires, analysis including all PPAs, k = 5). Example plot representing neuropsychological profiles for the analysis including patients diagnosed with one of the PPA variants, including questionnaires and limiting the analysis to k = 5 clusters. For the analysis scores were scaled to vary between -1 and 1. For visualization purposes only, cluster centers were z-standardized. Scores more at the inside of the radar plot correspond to worse performance. **(A)** Radar Plot indicating z-standardized cluster centers for clusters 1-5. **(B)** Box plot indicating groups within clusters 1-9. Percentages displayed in the plot correspond to within-cluster proportions and were annotated for the largest group within a cluster.

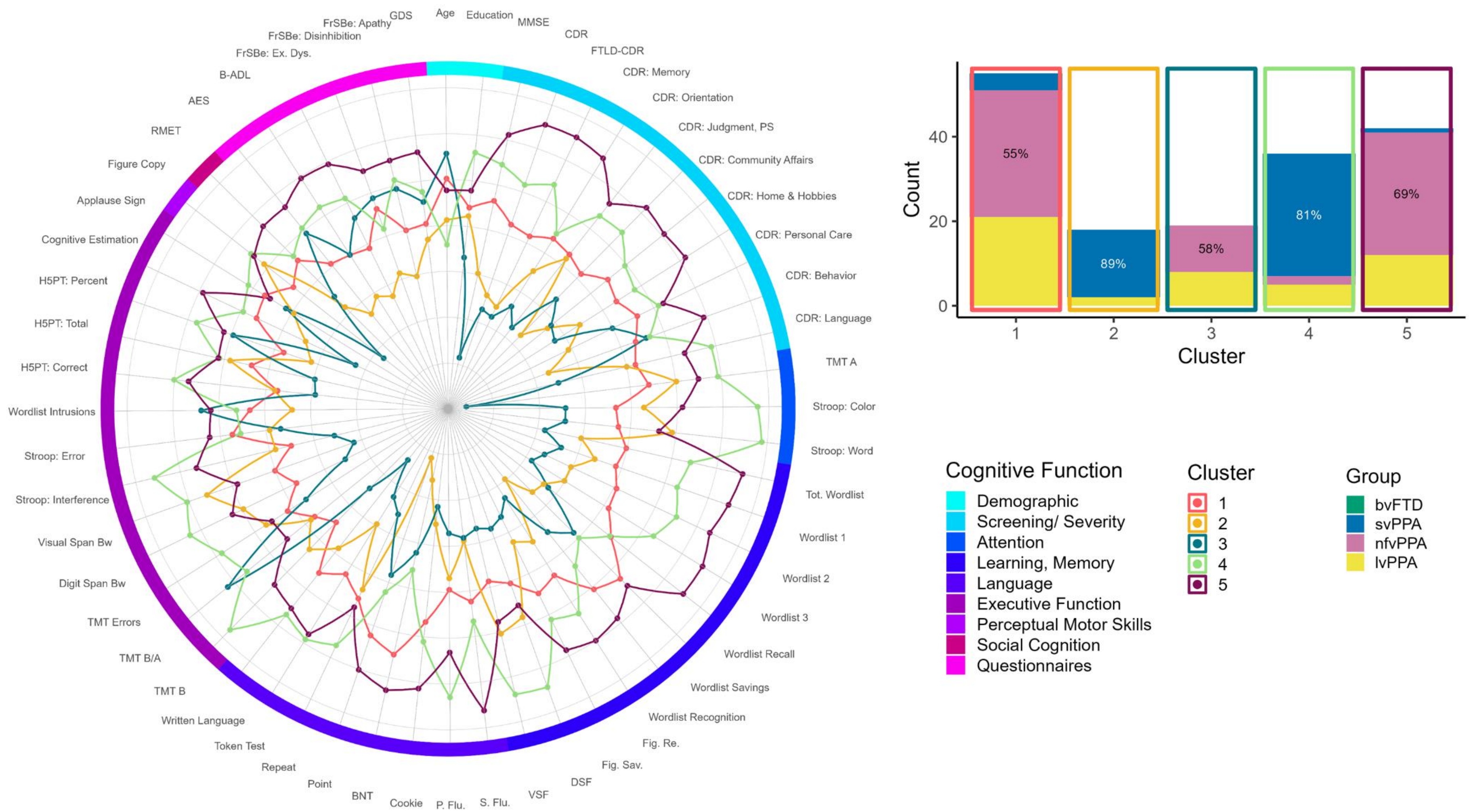

Abbreviations: AES Apathy Evaluation Scale, B-ADL Bayer Activities of Daily Living, BNT Boston Naming Test, CDR Clinical Dementia Rating Scale, DSF Digit span forward, Ex. Dys. Executive Dysfunction, Fig. Re. Figure Recall (from CERAD battery), Fig. Sav. Figure savings (from CERAD battery), FrSBe Frontal Systems Behavior Scale, FTLD-CDR Frontotemporal Lobar Degeneration Clinical Dementia Rating Scale, GDS Geriatric Depression Scale, H5PT Hamasch five point Test, LvPPA logopenic variant primary progressive aphasia, MMSE Mini-Mental State Examination, NfvPPA non-fluent variant primary progressive aphasia, TMT Trail Making Test, P. Flu.

Phonematic fluency, RMET Reading the mind in the eyes test, S. Flu. Semantic fluency, SvPPA semantic variant primary progressive aphasia, VSF visual span forward.

Figure S14

Neuropsychological profiles of cluster results (excluding questionnaires, analysis including bvFTD and amnestic AD, k = 4). Example plot representing neuropsychological profiles for the analysis including patients diagnosed with bvFTD and amnestic AD, excluding questionnaires and limiting the analysis to k = 4 clusters. For the analysis scores were scaled to vary between -1 and 1. For visualization purposes only, cluster centers were z-standardized. Scores more at the inside of the radar plot correspond to worse performance. **(A)** Radar Plot indicating z-standardized cluster centers for clusters 1-4. **(B)** Box plot indicating groups within clusters 1-9. Percentages displayed in the plot correspond to within-cluster proportions and were annotated for the largest group within a cluster.

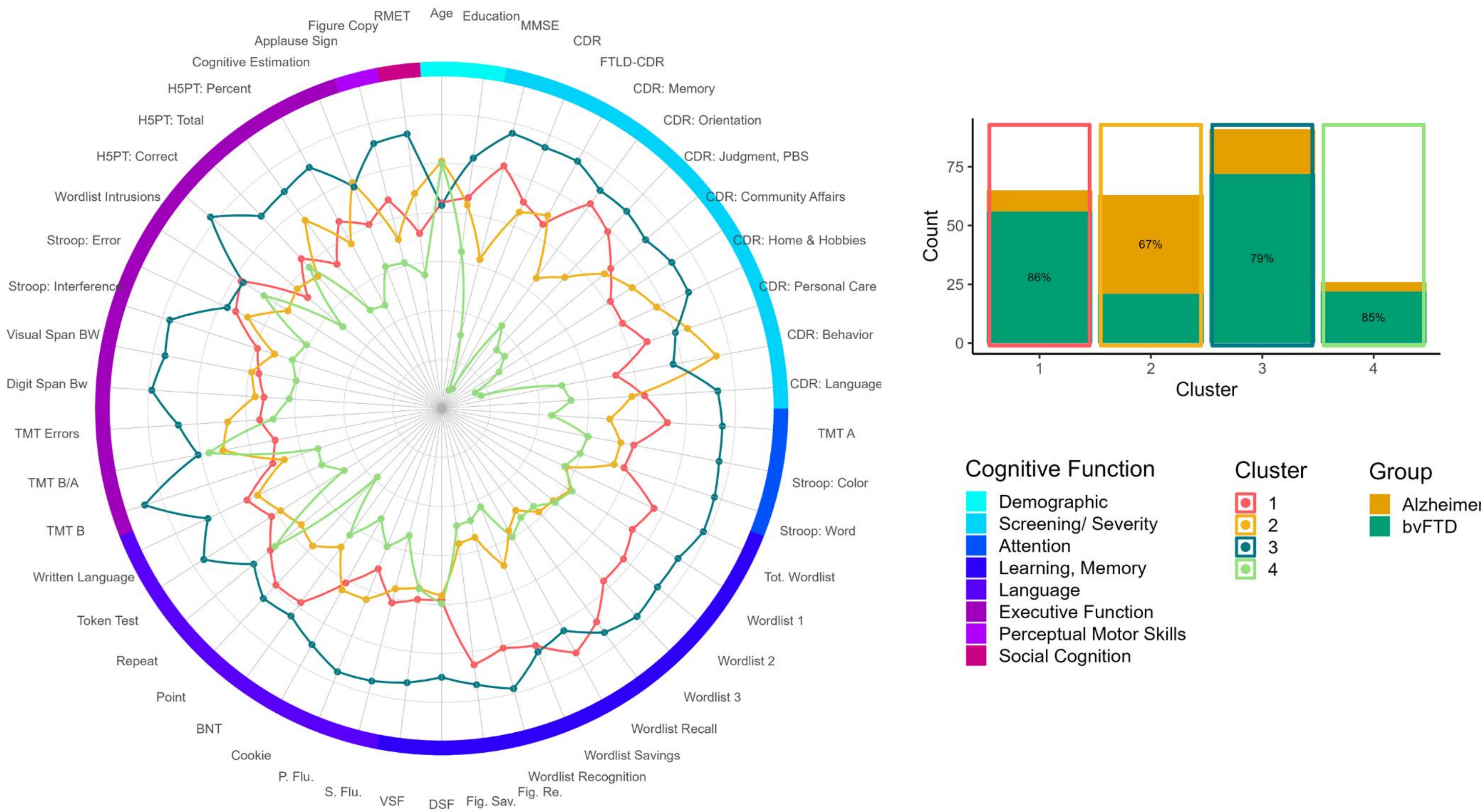

Abbreviations: AD amnestic Alzheimer's Disease, BNT Boston Naming Test, BvFTD behavioral variant frontotemporal dementia, CDR Clinical Dementia Rating Scale, DSF Digit span forward, Fig. Re. Figure Recall (from CERAD battery), Fig. Sav. Figure savings (from CERAD battery), FTLD-CDR Frontotemporal Lobar Degeneration Clinical Dementia Rating Scale, H5PT Hamasch five point Test, MMSE Mini-Mental State Examination, TMT Trail Making Test, P. Flu. Phonematic fluency, RMET Reading the mind in the eyes test, S. Flu. Semantic fluency, VSF visual span forward.

Figure S15

Neuropsychological profiles of cluster results (including questionnaires, analysis including bvFTD and amnestic AD, k = 3). Example plot representing neuropsychological profiles for the analysis including patients diagnosed with bvFTD and amnestic AD, including questionnaires and limiting the analysis to k = 3 clusters. For the analysis scores were scaled to vary between -1 and 1. For visualization purposes only, cluster centers were z-standardized. Scores more at the inside of the radar plot correspond to worse performance. **(A)** Radar Plot indicating z-standardized cluster centers for clusters 1-3. **(B)** Box plot indicating groups within clusters 1-9. Percentages displayed in the plot correspond to within-cluster proportions and were annotated for the largest group within a cluster.

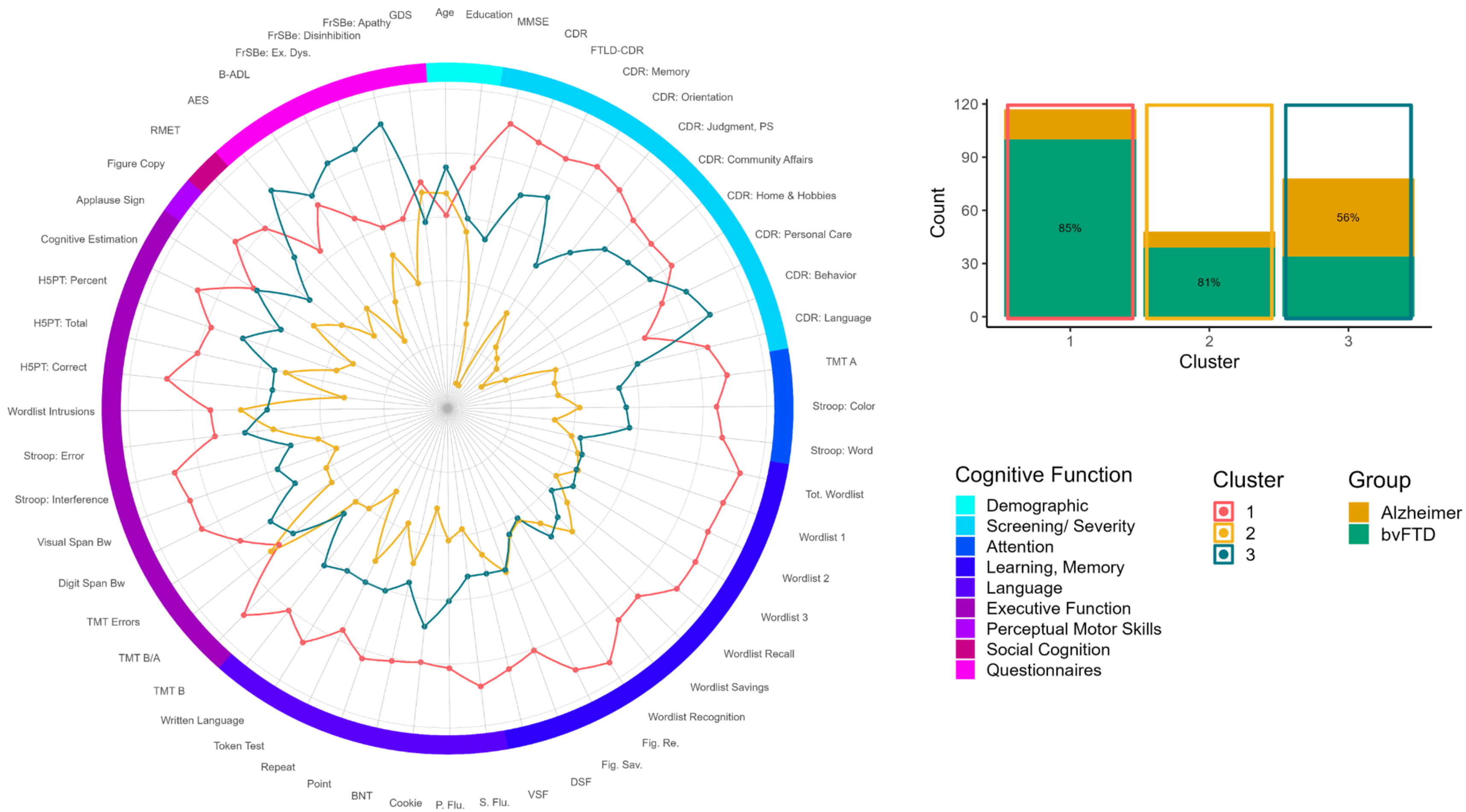

Abbreviations: AD amnestic Alzheimer's Disease, AES Apathy Evaluation Scale, B-ADL Bayer Activities of Daily Living, BNT Boston Naming Test, BvFTD behavioral variant frontotemporal dementia, CDR Clinical Dementia Rating Scale, DSF Digit span forward, Ex. Dys. Executive Dysfunction, Fig. Re. Figure Recall (from CERAD battery), Fig. Sav. Figure savings (from CERAD battery), FrSBe Frontal Systems Behavior Scale, FTLD-CDR Frontotemporal Lobar Degeneration Clinical Dementia Rating Scale, GDS Geriatric Depression Scale, H5PT Hamasch five point Test, MMSE Mini-Mental State Examination, TMT Trail Making Test, P. Flu. Phonematic fluency, RMET Reading the mind in the eyes test, S. Flu. Semantic fluency, VSF visual span forward.
